# The interaction of carbon nanotubes with an *in vitro* blood-brain barrier model and mouse brain *in vivo*

**DOI:** 10.1016/j.biomaterials.2015.02.083

**Published:** 2015-06

**Authors:** Houmam Kafa, Julie Tzu-Wen Wang, Noelia Rubio, Kerrie Venner, Glenn Anderson, Elzbieta Pach, Belén Ballesteros, Jane E. Preston, N. Joan Abbott, Khuloud T. Al-Jamal

**Affiliations:** a Institute of Pharmaceutical Science, King's College London, Franklin-Wilkins Building, 150 Stamford Street, London SE1 9NH, UK; b Institute of Neurology, University College London, Queen Square, London WC1N 3BG, UK; c Histopathology Department, Great Ormond Street Hospital for Children, London WC1N 3JH, UK; d ICN2 – Institut Catala de Nanociencia i Nanotecnologia, Campus UAB, 08193 Bellaterra, Barcelona, Spain

**Keywords:** Transcytosis, Transwells, PBEC, BBB model, TEM, STEM

## Abstract

Carbon nanotubes (CNTs) are a novel nanocarriers with interesting physical and chemical properties. Here we investigate the ability of amino-functionalized multi-walled carbon nanotubes (MWNTs-NH_3_^+^) to cross the Blood-Brain Barrier (BBB) *in vitro* using a co-culture BBB model comprising primary porcine brain endothelial cells (PBEC) and primary rat astrocytes, and *in vivo* following a systemic administration of radiolabelled *f*-MWNTs. Transmission Electron microscopy (TEM) confirmed that MWNTs-NH_3_^+^ crossed the PBEC monolayer *via* energy-dependent transcytosis. MWNTs-NH_3_^+^ were observed within endocytic vesicles and multi-vesicular bodies after 4 and 24 h. A complete crossing of the *in vitro* BBB model was observed after 48 h, which was further confirmed by the presence of MWNTs-NH_3_^+^ within the astrocytes. MWNT-NH_3_^+^ that crossed the PBEC layer was quantitatively assessed using radioactive tracers. A maximum transport of 13.0 ± 1.1% after 72 h was achieved using the co-culture model. *f*-MWNT exhibited significant brain uptake (1.1  ±  0.3% injected dose/g) at 5 min after intravenous injection in mice, after whole body perfusion with heparinized saline. Capillary depletion confirmed presence of *f*-MWNT in both brain capillaries and parenchyma fractions. These results could pave the way for use of CNTs as nanocarriers for delivery of drugs and biologics to the brain, after systemic administration.

## 1 Introduction

Carbon nanotubes (CNT) are novel nanomaterial with attractive physical, chemical and electronic properties [1]. One of the most interesting characteristics of functionalized CNTs (*f*-CNTs), from a drug delivery point of view, is their ability to translocate across plasma membranes and enter the cells either passively by direct translocation across membranes or actively *via* endocytosis [2]. Nano-meshworks of single-walled carbon nanotubes (SWNTs) and MWNTs on glass slides have been shown to support neuronal growth and responsiveness of rat hippocampal neurones. CNTs improved the excitability of the neurones by interfacing with the cells creating circuit shortcuts [3]. CNTs have also shown intrinsic therapeutic action in stroke prevention *in vivo* without carrying a therapeutic cargo. Lee et al. have studied the intrinsic therapeutic effect of amino-functionalized SWNTs in stroke prevention. A cerebral ischemia injury rat model was used in this study, with SWNTs injected into the lateral ventricle of the brain. The extent of neuronal damage following the ischemic injury was significantly lower in the SWNT-treated group compared to the control. The therapeutic effect persisted for 7 days after treatment showing the potential of SWNTs in stroke prevention [4]. Moreover, we have previously demonstrated the successful delivery of caspase-3 siRNA by CNTs to the brain *in vivo*. MWNTs-NH_3_^+^ were complexed with caspase-3 siRNA to provide a delivery platform superior to the intracerebral administration of siRNA alone. The peri-lesional stereotactic administration of MWNTs-NH_3_^+^:caspase-3 siRNA complexes resulted in reduced neuronal damage and enhanced motor function recovery in an Endothelin-1 rat stroke model [5]. In another study, the delivery of CpG oligonucleotide to brain glioma was enhanced using SWNTs. SWNTs-CpG conjugates were injected into the brain using a single intracranial injection and the uptake was evaluated using flow cytometry and confocal microscopy. The data suggested that conjugating CpG to SWNTs significantly enhanced the delivery into tumour-associated inflammatory cells compared to the free CpG [6]. These studies highlight the importance of using CNTs with/without therapeutic cargo to reach targets in the brain and eradicate tumours. However, none of the mentioned studies used systemic administration where crossing the BBB may constitute a major obstacle to CNTs brain delivery.

CNTs have shown the ability to permeate through biological membranes with evidence of membrane sealing due to their unique needle-like structure [7]. However, no studies on their ability to cross the BBB *in vitro* or *in vivo* have been reported. Therefore, in this work we hypothesize that MWNTs-NH_3_^+^, used as a prototype, are able to cross the PBEC cells *in vitro*, and accumulate in the brain following intravenous injection. The mechanism of BBB crossing was studied *in vitro*, using a co-culture model of porcine brain endothelial cells (PBEC) and primary rat astrocytes. MWNTs-NH_3_^+^ were shown to be able to cross the PBEC layer as confirmed by transmission electron microscopy (TEM) and gamma counting. *f*-MWNT were also found in the astrocyte layer of the co-culture. Brain uptake and access to mouse brain parenchyma, after systemic injection, were confirmed by gamma counting and capillary depletion, respectively.

## 2 Materials and methods

### 2.1 Materials

Newborn Calf heat-inactivated Serum and Bovine Plasma-Derived Serum were obtained from First-Link, UK Ltd. Iscove's Modified Dulbecco's Medium (IMDM), High glucose Dulbecco's Modified Eagle's Medium (DMEM), Low glucose DMEM, Medium 199, Phosphate Buffered Saline (PBS), 10×, pH 7.4, Minimum Essential Medium/HEPES, Penicillin-Streptomycin 100X, 0.05% Trypsin-EDTA (1X) with Phenol Red, GlutaMAX™ Supplement were obtained from Invitrogen, Life Sciences, UK. Collagenase type 3, Trypsin powder, Deoxyribonuclease I were obtained from Worthington Biochemicals Inc, USA. Nylon mesh 60 μm and 150 μm pore size, 50 mm disks were purchased from PLASTOK, UK. Sodium chloride, Trypsin solution from porcine pancreas, Cytosine arabinoside, Poly-*l*-lysine, Hanks' Balanced Salt solution (HBSS), Paraformaldehyde, HEPES, N,N-Dimethylformamide, 8-(4-Chlorophenylthio)adenosine 3′,5′-cyclic monophosphate sodium salt, 4-(3-Butoxy-4-methoxybenzyl)imidazolidin-2-one, Hydrocortisone, Dounce tissue grinder set, Fibronectin from bovine plasma, Propylene oxide, Uranyl acetate dihydrate, N,N-Diisopropylethylamine, Diethyl enetriamine pentaacetic acid, Ninhydrin, Dimethyl sulfoxide, Heparin sodium salt from porcine intestinal mucosa, Puromycin dihydrochloride from *Streptomyces alboniger*, Triton™ X-100, Osmium tetroxide, Costar Transwell™ permeable supports 3.0 μm pore polyester and polycarbonate membrane were all obtained from Sigma–Aldrich, UK. Fluoropore PTFE hydrophobic 0.22 μm membrane and Glutaraldehyde, 25% Aqueous Solution were purchased from Merck Millipore, UK. Precision Bottle-top filter unit MF75 Series disposable SFCA membrane and 70 μm cell Strainer were obtained from BD Falcon™, UK. Dodecenyl Succinic Anhydride, Araldite CY212, Tris(dimethylaminomethyl)phenol, F2 Finder Grids Copper 3.05 mm were obtained from AgarScientific, UK. Ultra Diamond Knife 45° was obtained from DiATOME, UK. MiniSart hydrophilic syringe filters (0.2 μm) were purchased from Sartorius Stedim UK Ltd. Pristine multi-walled carbon nanotubes, used for preparation of MWNTs-NH_3_^+^ (diameter = 20–30 nm, Length = 0.5 μm), Batch # 1237YJS were purchased from Nanostructured and Amorphous materials Inc. CytoTox 96^®^ Non-Radioactive Cytotoxicity Assay was obtained from Promega Corporation, UK. Sodium cacodylate trihydrate and Optiphase Trisafe scintillation cocktail were purchased from ThermoFisher Scientific. Sucrose [U-^14^C specific activity > 350 mCi/mmol ] 1.85 MBq was obtained from MP Biomedicals. The radioactive tracer [^111^In]Cl_3_ was obtained from Amersham Pharmacia Biosciences as an aqueous solution and used without further purification.

### 2.2 Functionalization of MWNTs derivatives

MWNTs constructs (MWNTs-NH_3_^+^, DTPA-MWNTs) were synthesized following previously published methods [5,8]. Detailed description of the synthesis methodology can be found in the SI Text. The synthesis of Boc-protected amino acid is described in Refs. [8,9]. The synthesized MWNTs were characterized by Kaiser test and thermogravimetric analysis. All details are provided in SI Text.

### 2.3 Radio-labelling of DTPA-MWNTs with ^111^Indium

DTPA derivatives of *f*-MWNT (1 mg/ml in water) were diluted with an equal volume of ammonium acetate buffer (0.2 M, pH 5.5) yielding a final acetate buffer of 0.1 M, pH 5.5, to which ^111^InCl_3_ was added. The reaction was carried out for 30 min at room temperature, and it was then terminated by the addition of EDTA quenching solution (0.1 M, 1:20 of total volume) to chelate the unreacted indium. The labelling efficiency of [^111^In]DTPA-MWNTs was determined by thin layer chromatography (TLC). Briefly, a small volume of [^111^In]DTPA-MWNTs was diluted 5 times in PBS, and 1 μl was applied on silica gel impregnated glass fibre sheets (Varian, USA). The TLC was carried out in 0.1 M ammonium acetate mobile phase containing 50 mM EDTA, and allowed to dry before counting the signal using the Cyclone phosphor detector (Packard Biosciences, UK). The spot at the application point of the TLC strip indicated the presence of [^111^In]DTPA-MWNTs whereas the signals at the solvent front were indicative of the [^111^In]EDTA and/or [^111^In]DTPA. The [^111^In]DTPA-MWNTs dispersions were also checked for [^111^In]DTPA contamination, as residual trace from DTPA chemical reaction, by TLC using 3.5% NH_4_^+^:methanol (1:1) as the mobile phase. In this case, the signals acquired at the application point were the result of the immobile [^111^In]DTPA-MWNTs or the colloidal indium (forms in basic conditions) whereas the signals at the solvent front indicated the presence of [^111^In]DTPA in the mixture. The stability of the [^111^In]DTPA-MWNTs formulation was tested by mixing with equal volume of PBS or serum and incubated at 37 °C for 24 h after radiolabelling. Any unbound indium was removed by a centrifugation step prior to incubation with cells. TLC with 0.1 M ammonium acetate mobile phase containing 50 mM EDTA was used to confirm the stability of the constructs during the time course of the experiment by taking samples from the apical and basal chambers. [^111^In]EDTA was used as a control.

### 2.4 The modified lactate dehydrogenase (LDH) assay

PBEC were seeded into a 24 well plate (2 × 10^4^ cells per cm^2^) and allowed to reach confluence. The culture medium was changed to serum-free medium 24 h before adding the MWNTs-NH_3_^+^ to the cells. The MWNTs-NH_3_^+^ dispersions were prepared in 5% dextrose solution, and then added onto the cells at 20 μg/ml and 50 μg/ml final concentrations. After 24 and 72 h, light microscopy images were captured. The cells were then washed twice with HBSS, and then lysed with Triton X-100 (0.09% v/v in fresh DMEM media). The cell lysates were collected and centrifuged for 15 min at 20,800 *g* (Eppendorf 5810R, Germany). The LDH content was assayed following the CytoTox 96^®^ assay protocol (Promega Corporation, USA), and the signal was measured at 490 nm using a FLUOstar Omega microplate reader (BMG Labtech, Germany).

### 2.5 Isolation of primary porcine brain endothelial cells (PBEC)

The PBEC isolation method was based on that originally developed for bovine brain endothelium by Rubin et al. [10] and was further modified and improved by Abbott and co-workers [11]. The porcine brains were delivered from the abattoir in ice-cold IMDM medium (IMDM supplemented with 100 U/100 μg/ml penicillin/streptomycin) and were kept on ice throughout the isolation. Brain hemispheres were washed briefly with ice-cold phosphate-buffered saline and cerebrums were removed. The meninges were peeled off with forceps from the surface of the brain and from inside the grooves, then the white matter was pinched off from inside the hemispheres. The cortices were collected in a sterile beaker containing MEM/HEPES medium (25 mM HEPES and 10% v/v FBS and 100 U/100 μg/ml penicillin/streptomycin) and chopped up with a sterile scalpel. For initial homogenization, the chopped hemispheres were forced through a 50 ml syringe into a T75 culture flask containing ice cold MEM/HEPES then further homogenized in a 40 ml Wheaton Dounce tissue grinder. The resulting homogenate was collected in a T-175 flask and filtered initially through a 150 μm nylon mesh then the filtrate was re-filtered through a 60 μm nylon mesh. The brain capillaries are trapped on the nylon meshes, which were then placed in a Petri dish containing the digest mix (collagenase type 3 (223 U/mg), trypsin (211 U/mg), DNase (2108 U/mg), FBS (10% v/v) and penicillin/streptomycin (100 U/100 μg/ml) dissolved in M199 medium) and incubated at 37 °C for 1 h. The brain capillaries were collected from the nylon meshes (capillaries collected on the 60 and 150 μm nylon meshes were kept separate) by trituration with digest mix and then centrifuged at 240 *g* (Eppendorf^®^ 5810R centrifuge, Germany) for 10 min. The supernatant was aspirated and the pellets were resuspended in 10 ml MEM/HEPES. This step was repeated twice and finally the pellets were resuspended in freshly-prepared freezing medium (FBS containing 10% v/v dimethyl sulfoxide). The suspension was then aliquoted in cryo-vials and stored at −80 °C overnight in a freezing container (1 °C per min freezing rate), and then in liquid nitrogen for long-term storage. PBEC 60's and 150's refer to brain capillaries collected on 60 and 150 μm meshes, respectively.

### 2.6 Culturing and maintenance of PBEC

To aid PBEC adhesion to cell culture vessels, the T-75 culture flasks were coated with 4 ml rat-tail collagen (330 μg/ml) in sterile de-ionized water for two hours at RT. Following the collagen coating, the flasks were washed twice with HBSS, 4 ml of fibronectin (7.5 μg/ml) was then added to each flask for 2 h at RT. After the cross-linking of collagen to fibronectin, the flasks were washed with HBSS. PBEC culture medium was freshly prepared to ensure that all the supplements were available to aid cell growth and differentiation. The optimum growth medium used for PBEC was low glucose DMEM supplemented with 10% v/v plasma-derived bovine serum, 100 U/100 μg/ml penicillin/streptomycin, 2 mM Glutamax™, 125 μg/ml heparin and 4 μg/ml puromycin. The medium was sterile-filtered using a 0.2 μm syringe filter. A vial of PBEC 60's was taken out of liquid nitrogen and allowed to thaw over 60 s in a 37 °C water bath. The vial was then taken into a sterile hood and diluted in 20 ml of warm PBEC medium. The suspension was then plated into the coated T-75 flasks. The medium was changed every 3 days until the culture was 50% confluent.

### 2.7 Isolation of primary rat astrocytes from post-natal rat pups

Primary astrocytes type I were isolated from 0 to 2 day old Wistar rat pups following the method of Abbott et al. [12]. Briefly, the brains were removed from the skulls aseptically in the hood and were kept in ice-cold dissection buffer (Ca^2+^ and Mg^2+^ free HBSS, 10 mM HEPES buffer, 100 U/ml penicillin G and 100 μg/ml streptomycin). The brains were dissected to remove the cerebrum, midbrain structures, choroid plexus and the hippocampus. The meninges were carefully removed and the cortices were placed in ice cold dissection buffer. The cortices were chopped finely with a sterile surgical scalpel, then an equal volume of trypsin solution (1.25 mg/ml) was added and the mixture was incubated at 37 °C for 30 min. Following the incubation period, the trypsin was neutralized by adding an equal volume of serum-containing nutrient medium (high glucose DMEM, 10% v/v fetal bovine serum, 1% Glutamax™, 100 U/ml penicillin G and 100 μg/ml streptomycin), and centrifuged for 5 min at 306 *g* (Beckman Coulter Allegra x-22R). The pellet was resuspended and triturated in 2 ml culture medium. The resulting homogenate was filtered through a 70 μm sieve and cells were counted on a haemocytometer. Cells were then seeded (25,000 cells/cm^2^) in poly-*l*-lysine (10 μg/ml) coated T25 flasks. The cultures were incubated at 37 °C in a humidified chamber with 5% CO_2_ and the culture medium was changed 24 h after seeding, and then every other day. At sub-confluence (7–9 days after seeding) the contaminating cells were eliminated by shaking the flasks on an orbital shaker for 24 h. Further purification was carried out at confluence by adding cytosine arabinoside (10 μM) to the flasks in the morning and replacing the culture medium in the evening for 5 days.

### 2.8 Setting up the BBB co-culture Transwell™ system

One week before the experiment, astrocytes were sub-cultured into a 12-well plate coated with 10 μg/ml poly-*l*-lysine. The medium was changed one day before introducing the Transwell™ filters in the well plate. A vial of PBEC 60's was thawed into two T-75 flasks, and the growth rate was monitored after three days. When the cells reached 50% confluency, they were sub-cultured onto 3.0 μm pore polyester inserts and placed in the wells above the astrocytes. The cells were incubated at 37 °C in a humidified chamber with 5% CO_2_ until PBEC formed a monolayer of endothelial cells on the filter. To stimulate the formation of tight junctions between adjacent endothelial cells, the culture medium was changed to serum-free medium for 24 h before starting uptake studies (third day of co-culture) as described previously [11]. In addition, the apical and basal chambers were supplemented with pCPT-cAMP (250 μM), RO-20-1724 (17.5 μM) and hydrocortisone (550 nM). After 24 h, the trans-endothelial electrical resistance (TEER) was measured (EVOM epithelial voltohmmeter with STX2 electrode, World Precision Instruments, UK) to assess the tightness of the BBB model. Permeability experiments were performed when TEER values were >200 Ω cm^2^.

### 2.9 Transport of f-MWNT across the BBB by electron microscopy

A PBEC/astrocyte co-culture BBB model was set up as detailed above. *f*-MWNTs were added to the apical chamber of the inserts (20 μg/ml) to examine interaction between *f*-MWNT and the PBEC monolayer by ultra-structural imaging. The cellular uptake experiments were carried out for 4, 24 and 48 h at 37 °C. To study the energy-dependency of *f*-MWNT uptake into PBEC, the PBEC/astrocytes BBB model was pre-incubated at 4 °C for 30 min followed by the addition of *f*-MWNT (20 μg/ml) to the apical chamber, as above. The plate was then incubated at 4 °C for a further 4 h. All uptake experiments were terminated by fixing the PBEC monolayer and the astrocytes with 2.5% glutaraldehyde solution in 0.1 M cacodylate buffer for 1 h at RT.

### 2.10 Sample processing of the Transwell™ filters and astrocytes for electron microscopy imaging

Transwell™ filters containing PBEC and the astrocytes layer were fixed using 1% osmium oxide for the purpose of TEM/scanning TEM (STEM) examination. The filters were rinsed with 0.1 M cacodylate buffer, and de-hydrated in a series of dehydration steps using increasing concentrations of ethanol (70%, 90% and absolute ethanol) being incubated for 10 min at each concentration. Propylene oxide was added to the filters for 15 min, followed by the addition of a 1:1 mixture of propylene oxide: epoxy resin for 1 h. The filters were then embedded into epoxy resin blocks (Araldite CY212, DDSA and DMP30) and left in 60 °C oven for 3 days to polymerize. Ultrathin sections were cut using a diamond knife on a microtome (Leica Reichert Ultracut) and collected on copper 200-mesh grids. The grids were stained with uranyl acetate for examination by TEM (Philips CM12, FEI Tecnai G2 F20, USA)/and low voltage STEM (FEI Magellan 400L XRSEM, USA).

### 2.11 Electron microscopy imaging of processed Transwell™ filters

Bright field TEM images of the PBEC and astrocytes samples were acquired using a Philips CM12 operating at 80 kV; this accelerating voltage did not affect the structure of the *f*-MWNT in the examined samples. The objective aperture was used to image the samples. Low voltage STEM imaging was carried out on the FEI extreme high resolution Magellan 400L SEM running at 10–15 kV fitted with a second generation FEI retractable STEM detector. This detector has multiple segments that allow capture of different signals simultaneously. The central part of the detector collects electrons at a normal angle producing conventional bright field images. Scattered electrons collected with an annular segment around the central part of the detector produce annular dark field (ADF) images. ADF images feature an inverse contrast that allows resolution of high electron dense structures, such as carbon nanotubes and metal catalysts with more detail. High angle annular dark field (HAADF) images are produced from scattered electrons that are collected on an annular dark field detector at higher angles; the intensity in HAADF images is directly related to the atomic number of the elements within the sample [13]. Finally, high-resolution TEM (HRTEM) images and STEM images (reported in SI) were obtained using a FEI Tecnai G2 F20 TEM running at 120 kV.

### 2.12 Transport studies of [^111^In]DTPA-MWNTs and [^14^C]sucrose across PBEC

The PBEC/astrocyte co-culture model was set up in a 12-well plate. [^111^In]DTPA-MWNTs (20 μg/ml) was added to the apical chamber of the Transwell™ inserts, and the plate was incubated at 37 °C up to 72 h. To examine the effect of temperature on the transport, three inserts were initially incubated at 4 °C for 4 h, followed by re-incubation at 37 °C. To assess the potential damage of [^111^In]DTPA-MWNTs on the tight junction assembly, the permeability marker [^14^C]sucrose (2.4 × 10^5^ dpm) was added to the apical chamber of transwells™ (in the presence or absence of MWNTs). The free isotope [^111^In]EDTA was used as another control. To assess the permeability of [^111^In]DTPA-MWNTs across the membrane filter (3.0 μm pore size), experiments were run in parallel where the filters were treated with the above conditions in the absence of PBEC/astrocytes. At specified time points (1 min, 3 min, 5 min, 10 min, 15 min, 30 min, 45 min, 60 min, 120 min, 240 min, 1080 min, 2880 min and 4320 min), 0.5 ml aliquots were taken from the basal chamber and stored in counter vials. The medium in the basal chamber was replenished with warm medium to maintain sink conditions. ^111^In signal was quantified as counts per minute (CPM) using the 1282 CompuGamma CS Gamma Counter (LKB Wallac, USA). When^111^In decayed, ^14^C signal was quantified using liquid scintillation method (LS 6500 scintillation counter, Beckman Coulter, USA), after the addition of 4 ml of the OptiPhase liquid scintillation cocktail (ThermoFisher, UK) to each tube. Percentage uptake was calculated from the total dose added to each well. Results were expressed as mean ± SD (*n* = 3).

The apparent permeability coefficient (P_app_) was calculated using the following equation [14];

$$P_{app}=\frac{\Delta C_{R}}{\Delta t}\frac{V_{R}}{A*\;C_{0}}$$

*ΔC*_*R*_/*Δt* is the change in concentration in the receiving chamber over time. This value was taken as the slope of the linear correlation (concentrations vs time) from short time point intervals (3 min–60 min). *C*_*0*_ is the starting concentration in the donor chamber (CPM), *V*_*R*_ is the volume of the donor chamber (ml) and *A* is the surface area of the Transwell™ filter (cm^2^).

### 2.13 Tissue bio-distribution of [^111^In]DTPA-MWNTs in mice by γ-scintigraphy

All *in vivo* experiments were conducted under the authority of project and personal licences granted by the UK Home Office and the UKCCCR Guidelines (1998). Tissue biodistribution of [^111^In]DTPA-MWNTs was measured by γ-scintigraphy. Female C57/Bl6 mice aged 5–6 weeks were anaesthetized by isoflurane inhalation and intravenously (i.v.) injected with [^111^In]DTPA-MWNTs (50 μg, 0.5 MBq) in 100 μl PBS *via* a single tail vein injection. At 5 min, 30 min, 1 h, 4 h and 24 h after injection, a whole body perfusion was performed on animals using 25 ml of heparinized saline (1000 U/L) through the left ventricle of the heart in order to wash out residual or loosely bound [^111^In]DTPA-MWNTs from the blood vessels. All the major organs including skin, liver, spleen, kidneys, heart, lungs, muscle, bone, brain, stomach and intestine were then harvested post-mortem. Excised organs were weighed and the radioactivity was measured by γ-scintigraphy (LKB Wallac 1282 Compugamma, PerkinElmer). The results are expressed as % injected dose per organ (%ID, mean ± SD, *n* = 3) or % injected dose per gram tissue (%ID/g, mean ± SD, *n* = 3) and statistically analyzed using 1-way ANOVA.

### 2.14 Brain capillary depletion

Brain tissues were subjected to a further capillary depletion method to separate the parenchyma from brain capillaries. Brain tissue was placed in a glass homogenizer in 0.8 ml of ice cold depletion buffer (10 mM HEPES in HBSS, pH 7.4). The brain was homogenized with 15 stokes of the pestle, and 1.6 ml of depletion buffer containing 26% dextran (148 kDa) was added into the homogenizer. The brain was further homogenized with 3 strokes of the pestle. Brain homogenate was centrifuged at 3220 *g* for 15 min. Brain parenchyma (supernatant) and brain vasculature (pellet) were collected into scintillation vials and the activity was measured using gamma counting. The results are expressed as % injected dose per organ (%ID, mean ± SD, *n* = 3) and statistically analyzed using 1-way ANOVA.

## 3 Results

### 3.1 Chemical functionalization of MWNTs-NH_3_^+^

Pristine MWNTs were functionalized with 1, 3-dipolar cycloaddition (Scheme S1). The cycloaddition reaction on MWNTs was performed using a Boc-protected amino acid and formaldehyde. Following this reaction, the Boc-group was deprotected using acidic conditions. The introduction of functional groups to the sidewalls of the pristine material was confirmed by TGA and Kaiser test. Kaiser test is a quantitative test based on the reaction of the ninhydrin reagent with the free amine group [15]. Due to the potential aggregation of MWNTs-NH_3_^+^, not all amine groups may have reacted with the ninhydrin reagent rendering this test semi-quantitative in this instance. TGA on the other hand offers quantitative amine loading values by assessing the weight loss exhibited upon heating the sample to 1000 °C. TGA results are therefore considered more accurate to quantify amine loading. Fig. S1 shows the TGA results of the synthesized MWNTs. The pristine MWNTs exhibited high thermal stability up to 750 °C after which they started to decompose. In comparison, MWNT-NH_3_^+^ exhibited 9% mass loss at 600 °C, which corresponds to 529 μmol of amine groups per gram of MWNTs. Additionally, MWNT-NH_3_^+^ started to decompose at a temperature lower than the decomposition temperature (750 °C) of the pristine material, which also indicated the conjugation of functional groups to the MWNTs. This data was further confirmed using Kaiser test to assess the amount of amine loading on the MWNTs. Fig. S2A shows the UV–vis spectrum of the MWNT-NH_3_^+^ after the Ninhydrin reaction. The spectrum showed an absorbance peak at λ = 575 nm indicating the presence of amine groups on the sidewalls of MWNTs.

DTPA-MWNTs were synthesized from MWNT-NH_3_^+^ as detailed in the SI Text. The ammonium groups of the MWNT-NH_3_^+^ were neutralized using Et_3_N to initiate the reaction with DTPA. The conjugation of DTPA to the amine groups was confirmed by the negative Kaiser test (Fig. S2A). DTPA-MWNTs (Fig. S1) showed 26% mass loss at 600 °C by TGA corresponding to 493 μmol of DTPA groups per gram of MWNTs. The above results indicated the successful conjugation of DTPA to MWNT-NH_3_^+^.

**Fig. 1:**
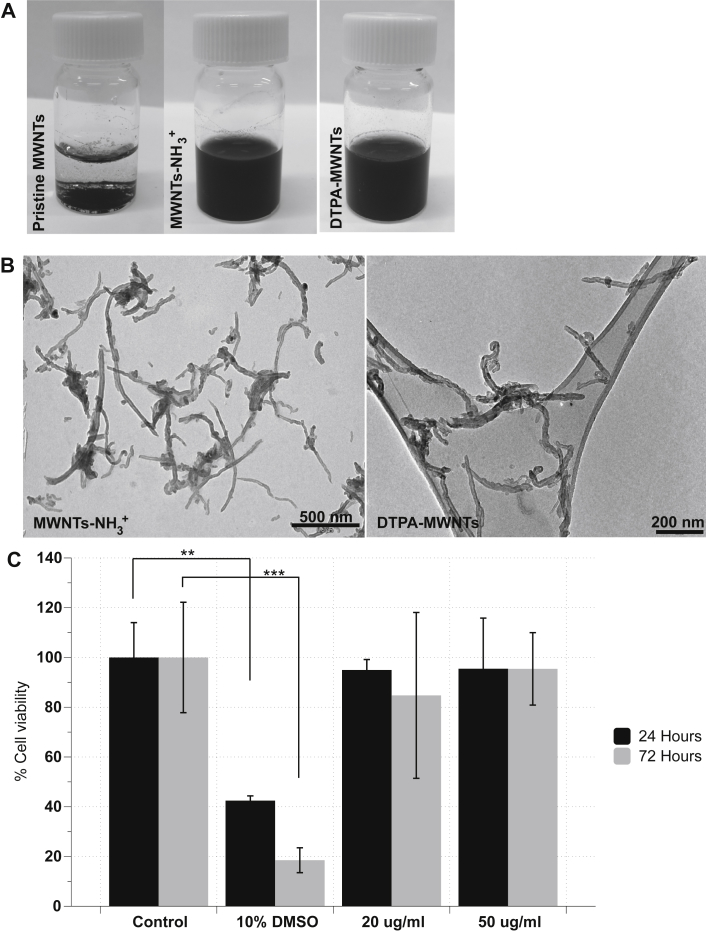
**The dispersibility of *f*-MWNTs constructs and the toxicity of MWNTs-NH**_**3**_^**+**^**on PBEC after 24 and 72 h of uptake**. The water dispersibility of the functionalized MWNTs-NH_3_^+^ and DTPA-MWNTs in water as observed visually (**A**) and by TEM examination (**B**). (**C**) The modified LDH assay was carried out to evaluate cell viability after 24 and 72 h of incubation at 20 and 50 μg/ml. 10% DMSO (v/v) was used as positive control. MWNTs-NH_3_^+^ had minimal effect on cell viability after 24 and 72 h while the viability of the cells treated with 10% DMSO was significantly reduced compared to the control at both time points. MWNTs-NH_3_^+^ did not affect the viability of PBEC up to 50 μg/ml (one-way ANOVA test; *p < 0.05, **p < 0.01, ***p < 0.001, n = 3).

Fig. 1A shows the improvement in MWNTs-NH_3_^+^ and DTPA-MWNTs aqueous dispersibility compared to that of the pristine material. TEM images confirmed that both constructs appeared well dispersed in water (Fig. 1B). The length and diameter distribution of MWNT-NH_3_^+^ is shown in Fig. S2B. The median of the length and diameter was 500 nm and 18.9 nm, respectively.

### 3.2 Assessing the toxicity of MWNTs-NH_3_^+^ on PBEC

The modified LDH assay was used to assess the potential cytotoxicity of MWNTs-NH_3_^+^ on PBEC relying on the percentage of LDH enzyme remaining within the cell reflecting viability of the cells [16] as we previously reported [17]. Dimethyl sulfoxide (DMSO) (10% v/v) was used as a positive control in this experiment. Two concentrations of MWNTs-NH_3_^+^ were tested: 20 μg/ml and 50 μg/ml. The culture medium was changed to serum-free medium 24 h before starting the experiment to mimic the conditions of the transport experiment.

The modified LDH assay (Fig. 1C) showed that MWNTs-NH_3_^+^ caused no statistically significant toxicity (*p* > 0.05) on the endothelial cells at the tested concentrations after 24 and 72 h. However, the viability of the cells treated with 10% DMSO, used as a positive control, was significantly lower than that of the control at 24 h (*p* < 0.01) and 72 h (*p* < 0.001). This reduction in viability confirmed the validity of the LDH assay to assess the viability of PBEC after incubation with MWNTs-NH_3_^+^. The lack of toxicity proved that MWNTs-NH_3_^+^ are safe to use in primary endothelial cell uptake studies under the conditions described. Despite the absence of toxic effects of MWNTs-NH_3_^+^ on the viability of PBEC, the lowest concentration (20 μg/ml) was used in all transport uptake experiments.

### 3.3 The interaction of MWNTs-NH_3_^+^ and PBEC monolayer by low voltage scanning transmission electron microscopy (STEM)

The first evidence of the interaction between *f*-MWNTs and PBEC was obtained from light microscopy imaging. Bright field light microscopy images confirmed the interaction between the MWNTs-NH_3_^+^ and the endothelial cells after 24 and 72 h incubation (Fig. S3A, B). The interaction between MWNTs-NH_3_^+^ and PBEC was evident after 24 h, where MWNTs-NH_3_^+^ appeared associated with the endothelial cells. After 72 h, the interaction between MWNTs-NH_3_^+^ and PBEC was more prominent. Even though the MWNTs-NH_3_^+^ appeared to accumulate within the cell after 24 and 72 h in a concentration-dependent manner, light microscopy was not sufficient to confirm the extent of uptake at the tested time points.

To investigate the ability of *f*-MWNTs to cross the PBEC monolayer, we used a co-culture BBB model of primary porcine endothelial cells and primary astrocytes. The BBB model was established in a Transwell™ system. The primary astrocytes were grown at the bottom of the culture plate and Transwell™ filters were then inserted into the well plates. Co-culturing endothelial cells with astrocytes has previously been shown to improve the polarized BBB phenotype of brain endothelial cells in culture, especially important for complex processes such as transcytosis [18,19]. PBEC was allowed to form a monolayer on a porous polyester filter creating the main BBB interface that is supported by mediators released from the primary astrocytes. Depriving the cells of serum enhanced the formation of tight junctions and allowed the polarization of endothelial cells in response to the growth factors for the primary astrocytes [11,20]. TEER measurements were carried out to monitor the tightness of the PBEC monolayer. TEER was measured after 24 h of serum deprivation (241.79 ± 27.76 Ω cm^2^, *n* = 8).

**Fig. 2:**
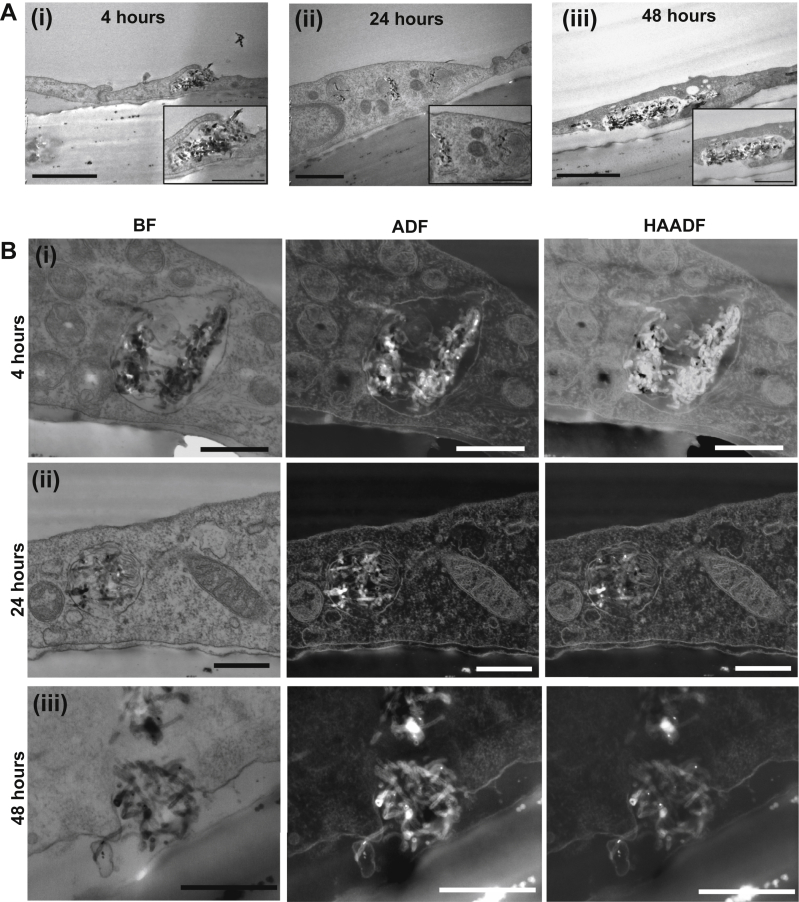
**The transcytosis pattern of MWNTs-NH**_**3**_^**+**^**across the PBEC monolayer following the incubation of MWNTs-NH**_**3**_^**+**^**with an *in vitro* BBB model**. MWNTs-NH_3_^+^ (20 μg/ml) were added to the apical chamber and incubated with the PBEC for (i) 4, (ii) 24 and (iii) 48 h. (**A**) Bright field TEM images of polyester filters showing the initial interaction of MWNTs-NH_3_^+^ with the PBEC monolayer after 4 h (i). After 24 h (ii), the MWNTs-NH_3_^+^ clusters appeared within endocytic vesicles or multi-vesicular bodies. The MWNTs-NH_3_^+^-containing vesicles showed evidence of fusion with the abluminal plasma membrane, and were partly open towards the basal chamber After 48 h (iii). (**B**) Low voltage STEM images of the polyester filters confirming the uptake and transcytosis of MWNTs-NH_3_^+^ across the PBEC monolayer. MWNTs-NH_3_^+^ appeared within endocytic vesicles after (i) 4 and (ii) 24 h. The MWNTs-NH_3_^+^-containing vesicles were then imaged partly open towards the basal chamber allowing the release of MWNTs-NH_3_^+^ after 48 h (iii). The three detection modes: bright field (BF), annular dark field (ADF) and high angular annular dark field (HAADF) helped identifying the electron dense structures of MWNTs-NH_3_^+^ within the cell body, and the osmium-rich lipid membranes surrounding the nanostructures. Scale bars: (A) 1 μm and (inset) 500 nm. (B) First and second row 500 nm and last row 400 nm.

TEM images (Fig. 2Ai) show the interaction of MWNTs-NH_3_^+^ and the plasma membrane of the endothelial cells after 4 h of incubation. MWNTs-NH_3_^+^ were able to interact with the plasma membrane of PBEC and triggered the formation of lipid ruffles that initiated the endocytosis sequence to engulf the MWNTs-NH_3_^+^, as a first step in transcytosis. After 24 h of incubation, most of the MWNTs-NH_3_^+^ were observed within endoplasmic vesicles (EVs) and multi-vesicular bodies (MVBs) (Fig. 2Aii, Figs. S4 and S5A). This vesicular uptake allowed the clusters of MWNTs-NH_3_^+^ into the PBEC monolayer as a second step in the transcytosis sequence after interaction with the plasma membrane occurred. The TEM images captured at 24 and 48 h confirmed the third step of the transcytosis process (Fig. 2Aiii and Fig. S4). Some MWNTs-NH_3_^+^ were found on the basal side of PBEC or within partly open vesicles facing the basal chamber at 24 h (Fig. S5). This provided the first evidence of the complete crossing of the endothelial cell *via* transcytosis. The data obtained here show for the first time that chemically functionalized MWNTs-NH_3_^+^ are able to cross the BBB *in vitro*.

To gain a better understanding of the behaviour of MWNTs-NH_3_^+^ within the endothelial cells, low voltage STEM imaging was employed as an advanced imaging method allowing the capture of high-resolution images of the MWNTs-NH_3_^+^ within PBEC. For this purpose, an extremely high resolution SEM fitted with a STEM detector was used. STEM images, especially ADF and HAADF, provided extra information regarding the transcytosis of MWNTs-NH_3_^+^. All the imaged sections were stained with osmium tetroxide and uranyl acetate, resulting in high contrast images of cellular lipid membrane and sub-cellular structures due to the high atomic number of osmium [21]. Therefore, ADF and HAADF images reflected the state and integrity of the plasma membrane and vesicular membranes during the transcytosis stages of MWNTs-NH_3_^+^. They also showed the behaviour of the highly electron dense MWNTs-NH_3_^+^ within PBEC. The MWNTs-NH_3_^+^ were observed in EVs after 4 and 24 h (Fig. 2Bi and Bii). After 48 h, MWNTs-NH_3_^+^ clusters were observed in partly open vesicles fused with the abluminal plasma membrane with some MWNTs-NH_3_^+^ exiting the PBEC (Fig. 2Biii) matching the data of the conventional TEM. The vesicles after 48 h appeared larger (1049 ± 222.3 nm, *n* = 5 cells) than those after 24 h (712 ± 106.1 nm, *n* = 5 cells), which may indicate that multiple vesicles fused together to form this large structure. The MWNTs-NH_3_^+^ were not observed within the tight junctions during the uptake process, which suggested that MWNTs-NH_3_^+^ were solely dependent on the transcellular route to cross the BBB, and they were not able to cross paracellularly.

The intact tight junctions suggested that the transcytosis of MWNTs-NH_3_^+^ has no impact on BBB integrity. This is very important from a toxicological and drug delivery point of view as it shows the ability of MWNTs-NH_3_^+^ to cross the BBB *in vitro* without affecting the permeability and healthy function of the BBB. MWNTs-NH_3_^+^ were mainly observed in EVs and MVBs, but were not spotted within other sub-cellular structures. The mitochondria appeared free of any interaction with MWNTs-NH_3_^+^, which is vitally important for the healthy function of the endothelial cell. The interaction of MWNTs-NH_3_^+^ with specific sub-cellular targets i.e. EVs or MVBs indicates that the MWNTs-NH_3_^+^ were following a specific trafficking pathway within the endothelial cells. This observation is important as the MWNTs-NH_3_^+^ used in this study were not functionalized with surface ligands for specific receptors, yet the uptake appears to take place in an organized and specific manner. ADF and HAADF images (Fig. S5Aiii and Bii) showed that upon release of MWNTs-NH_3_^+^ from PBEC, the vesicular membrane was intact and complete at the edges of the releasing vesicle. This observation further confirmed that the release of MWNTs-NH_3_^+^ is regulated by the endothelial cell, and is not a damaging effect of the MWNTs-NH_3_^+^.

Besides MWNTs-NH_3_^+^, we examined the ability of MWNTs-COOH to cross PBEC cells *in vitro*, to determine whether the uptake is charge-dependent. Interestingly, all *f*-MWNT studied triggered vesicular uptake regardless of charge, indicating that *in vitro* BBB transcytosis was charge-independent (Fig. S6).

The stability of MWNTs-NH_3_^+^ following uptake into PBEC was studied using high resolution TEM (HRTEM) and Electron Energy Loss Spectroscopy (EELS). Fig. S7 showed no evidence of breaking down within the cells indicated by the preserved graphitic structure of the MWNTs-NH_3_^+^ within the vesicles. Further details on this part of results are provided in the SI Text.

### 3.4 The interaction of individual MWNTs-NH_3_^+^ with the PBEC monolayer

**Fig. 3:**
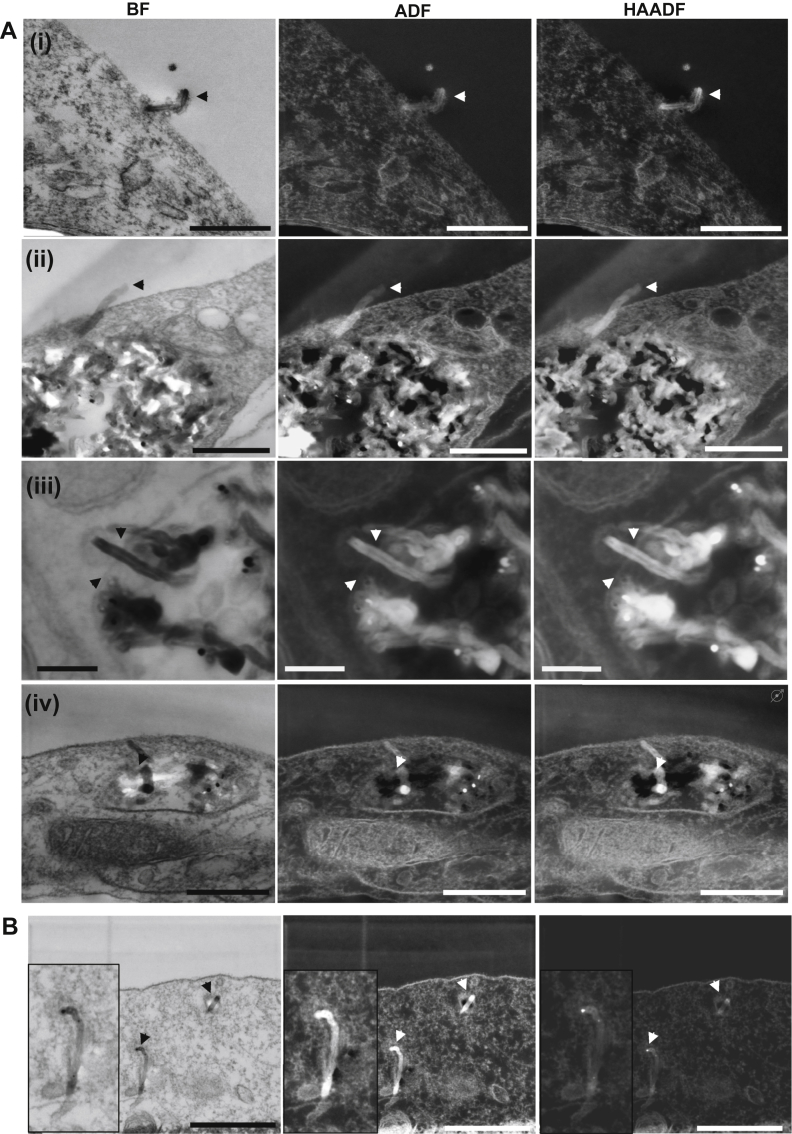
**Translocation of “individual” MWNTs-NH**_**3**_^**+**^**across the PBEC membrane**. Individual MWNTs-NH_3_^+^ shows an apparent ability to pierce through the plasma membrane. (**A**) Bright field STEM images showing a single MWNTs-NH_3_^+^ (black and white arrow heads) crossing the plasma membrane of endothelial cells (**i**, **ii**). The ADF and HAADF images clearly demonstrate part of the nanotube on the other side of the plasma membrane. The ability of MWNTs-NH_3_^+^ to permeate through plasma membranes was not only demonstrated at the plasma membrane level, but also seen as a transport method within the cell (**iii**, **iv**). (**B**) Electron micrographs showing a single nanotube that does not appear to be associated with other sub-cellular compartments. Close examination reveals the presence of a thin membrane around the single nanotube. Scale bars (A) from top to bottom, 400 nm, 300 nm and 100 nm, 300 nm. (B) 500 nm.

One of the advantages of using MWNTs-NH_3_^+^ as nanocarriers is their ability to cross biological membranes. The needle shape, yet flexible structure allows MWNTs-NH_3_^+^ to cross plasma membranes without utilizing the cellular endocytic processes. Many studies have established the ability of MWNTs-NH_3_^+^ to cross biological membranes by direct translocation in addition to active uptake mechanisms, but none of these studies have explored blood–brain barrier endothelial cells. Low voltage STEM imaging in the present study revealed that a small number of MWNTs-NH_3_^+^ were able to pierce the plasma membrane of endothelial cells within the PBEC monolayer. Fig. 3Ai, ii demonstrates this uptake pattern where MWNTs-NH_3_^+^ were shown to interact with the plasma membrane as individual carbon nanotubes, and appeared to be crossing this membrane without any sign of membrane invagination.

MWNTs-NH_3_^+^ have also been observed exiting the EVs within the PBEC monolayer. The MWNTs-NH_3_^+^ were able to pierce the membrane of the EVs to access the cytosol (Fig. 3Aiii, iv). It is possible that MWNTs-NH_3_^+^ were routed from the EVs. However, one cannot exclude the possibility that the MWNTs-NH_3_^+^ may have been entering the vesicle instead.

Fig. 3 shows that some individual MWNTs-NH_3_^+^ were able to move freely within the brain endothelial cells by crossing the cellular membranes. This ability provides a unique internalization route for MWNTs-NH_3_^+^ to evade the conventional transcytosis mechanisms used by other nanocarriers, which could be advantageous from a drug delivery point of view. A complete translocation of the plasma membrane by individual MWNTs was observed in only one instance (Fig. 3B). However, this single MWNTs-NH_3_^+^ was surrounded by a thin membrane, which may indicate that the MWNTs-NH_3_^+^ was taken into the endothelial cell by the active endocytic mechanism. Very few individual MWNTs-NH_3_^+^ were observed crossing the plasma membrane in our uptake studies compared to the large numbers of MWNTs-NH_3_^+^ entering the endothelial cells in vesicles. Therefore, the data presented thus far is insufficient to draw a conclusion regarding the uptake of individual MWNTs-NH_3_^+^, and more experiments are needed to understand this route of uptake, which has been described extensively for other cell types [22–24].

### 3.5 Uptake inhibition of MWNTs-NH_3_^+^ in PBEC

**Fig. 4:**
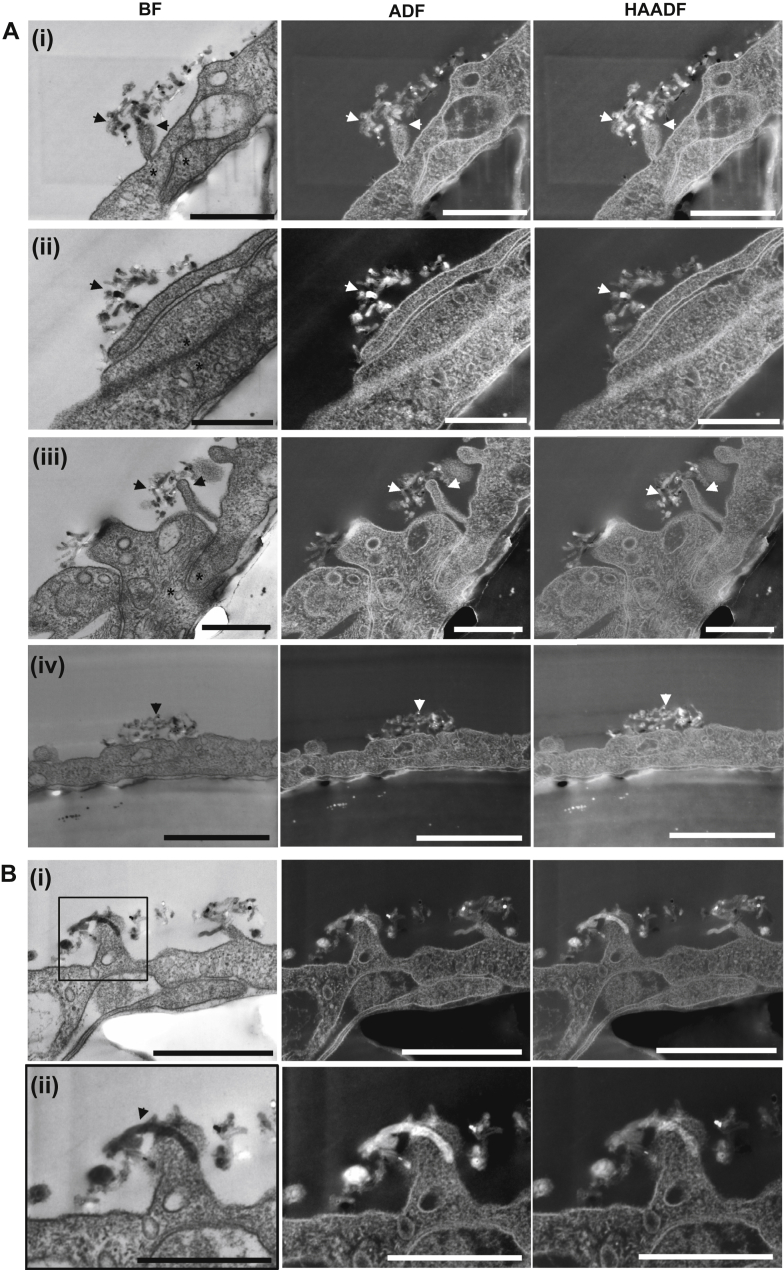
**Inhibition of MWNTs-NH**_**3**_^**+**^**uptake into endothelial cells following the incubation of MWNTs-NH**_**3**_^**+**^**at 4 °C**. (**A**) The transport experiment was carried out at 4 °C for 4 h to evaluate the energy dependency of MWNTs-NH_3_^+^ uptake into the endothelial cells. Low voltage STEM images show the accumulation of MWNTs-NH_3_^+^ outside the endothelial cells after 4 h of incubation, but no evidence of vesicular uptake was observed. The images confirmed that transcytosis of MWNTs-NH_3_^+^ was governed by an active mechanism. The MWNTs-NH_3_^+^ were mostly observed around endothelial extensions (arrow heads) in the vicinity of a tight-junctional zone (asterisks) (**i**–**iii**). (**B**) Electron micrographs showing a single MWNTs-NH_3_^+^ interacting with the endothelial extension at 4 °C (**i**, **ii**). Part of the MWNTs-NH_3_^+^ appeared on the other side of the plasma membrane. Scale bars (A) from top to bottom, 500 nm, 400 nm and 500 nm, 1 μm. (B) From top to bottom 500 nm and 300 nm.

At 37 °C, the entry of MWNTs-NH_3_^+^ into the PBEC monolayer occurred mostly *via* vesicular uptake with MWNTs-NH_3_^+^ clusters being transported from the apical to the basal side of the endothelial cell. To examine the effect of temperature on the transcytosis, MWNTs-NH_3_^+^ were incubated with the cells for 4 h at 4 °C to inhibit the energy-dependent route of uptake. Low voltage STEM imaging confirmed that incubating the cells with the MWNTs-NH_3_^+^ at 4 °C resulted in inhibition of vesicular uptake. Fig. 4, Ai–Aiv (black and white arrow heads) showed the accumulation of MWNTs-NH_3_^+^ at the apical side of the endothelial cells. As vesicle formation was inhibited at the low temperature, the MWNTs-NH_3_^+^ clusters were unable to enter the PBEC monolayer. This observation provides further evidence that MWNTs-NH_3_^+^ uptake is dependent on cellular endocytosis mechanisms. However, the MWNTs-NH_3_^+^ clusters that were unable to enter the cell were observed at sites adjacent to endothelial tight junctions showing some interaction with cellular extensions. To examine this phenomenon further, we measured the distance between the centre of the MWNTs cluster and the point of apical access to the tight junction (shown by asterisks). The distance was estimated to be (620.2 ± 152.5 nm, *n* = 4). Further studies need to be carried out to confirm the MWNTs-NH_3_^+^ specificity for the para-junctional zone.

A few individual MWNTs-NH_3_^+^ were able to pierce the plasma membrane during the 4 °C incubation. Fig. 4B shows an example of an individual MWNTs-NH_3_^+^ that was able to penetrate the plasma membrane. The piercing mechanism can therefore be considered as an energy-independent mechanism allowing MWNTs-NH_3_^+^ to permeate through lipid membranes independent of active uptake mechanisms. However, it appears to contribute only a small fraction of MWNTs-NH_3_^+^ cell entry. This suggested that some individual MWNTs-NH_3_^+^ can enter the cell in a passive energy-independent pathway.

### 3.6 MWNTs-NH_3_^+^ localization within astrocytes after crossing the BBB

**Fig. 5:**
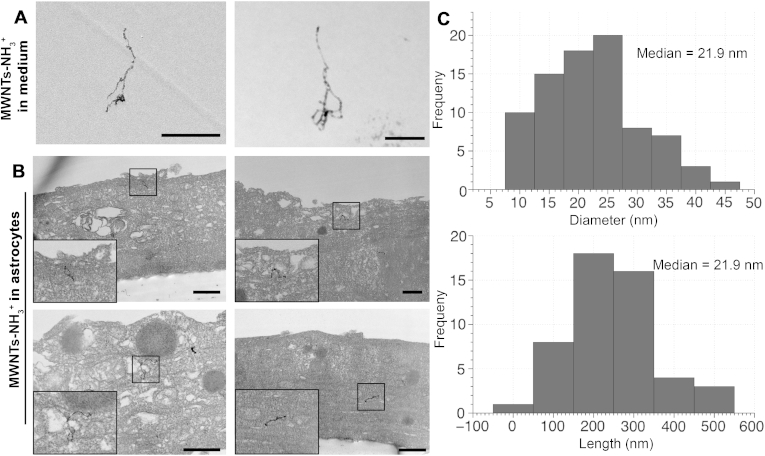
**Uptake of MWNTs-NH**_**3**_^**+**^**into primary astrocytes following PBEC transcytosis**. Electron micrographs showing the fate of MWNTs-NH_3_^+^ following the crossing of the PBEC monolayer. MWNTs-NH_3_^+^ were tracked into the basal chamber after 24 h of incubation with PBEC. (**A**) The structure of MWNTs-NH_3_^+^ in the medium of the basal chamber following crossing the PBEC/filter. (**B**) Electron micrographs showing the uptake of MWNTs-NH_3_^+^ into the primary astrocytes. Individual MWNTs-NH_3_^+^ were observed within the astrocytes with no evidence of vesicular uptake. (**C**) Length and diameter distribution histogram (*n* = 100) of the studied MWNTs-NH_3_^+^. The median length and diameter of the MWNTs-NH_3_^+^ was 233.9 nm and 21.9 nm, respectively. Scale bars = 500 nm.

The presence of MWNTs-NH_3_^+^ in the basal chamber, both in the medium and within the astrocytes, was investigated to further confirm the crossing of the PBEC monolayer. Fig. 5A shows the MWNTs-NH_3_^+^ that were found in the medium following the crossing of the PBEC monolayer and the open pores. Fig. 5B shows that following the crossing of the PBEC monolayer, MWNTs-NH_3_^+^ appeared to interact with the astrocyte layer and accumulated within the cell body. Individual MWNTs-NH_3_^+^ were observed within the astrocytes after 24 h, which further proves the ability of MWNTs-NH_3_^+^ to cross BBB *in vitro*. HAADF images were obtained to map the electron density of the observed structures and therefore confirm their nature. HAADF images (Fig. S8) show the structure of MWNTs in high contrast against the cell background indicating the high electron density, which is characteristic of the MWNTs-NH_3_^+^. HAADF images and their BF counterparts provided a direct confirmation of the nature of the nanomaterial in the astrocytes.

MWNTs-NH_3_^+^ were observed in the cytoplasm of astrocytes with no clear association with sub-cellular organelles. This indicates that MWNTs-NH_3_^+^ crossed the plasma membrane of astrocytes without any membrane invagination, which was different from that observed in PBEC. The ability of MWNTs-NH_3_^+^ to utilize multiple uptake routes into the cells, i.e., vesicular route of MWNTs-NH_3_^+^ clusters into PBEC and direct membrane crossing of individual MWNTs-NH_3_^+^ into astrocytes, shows the potential for using *f*-MWNTs to target multiple cell types *in vivo*. It is worth noting that the median length and diameter of the *f*-MWNTs (∼233.9 nm and ∼21.9 nm, respectively) (Fig. 5C) were comparable to that of the starting material (Fig. S2B). Overall, the presence of MWNTs-NH_3_^+^ within the astrocytes confirmed the ability of MWNTs-NH_3_^+^ to cross the BBB *in vitro*.

### 3.7 Transport of radiolabelled MWNTs-DTPA across the PBEC monolayer in an *in vitro* BBB model

DTPA-MWNTs constructs were labelled with ^111^Indium as detailed in the methods. Following the labelling reaction, the radiolabelling efficiency was measured by TLC. Fig. S9A shows labelling efficiency of [^111^In]DTPA-MWNTs to be 8.1%. The labelled constructs were tested for [^111^In]DTPA contamination by running the TLC using 3.5% NH_4_^+^:methanol (1:1) as the mobile phase. Fig. S9B confirms the presence of only negligible amount of free DTPA (4.4%) in the sample.

**Fig. 6:**
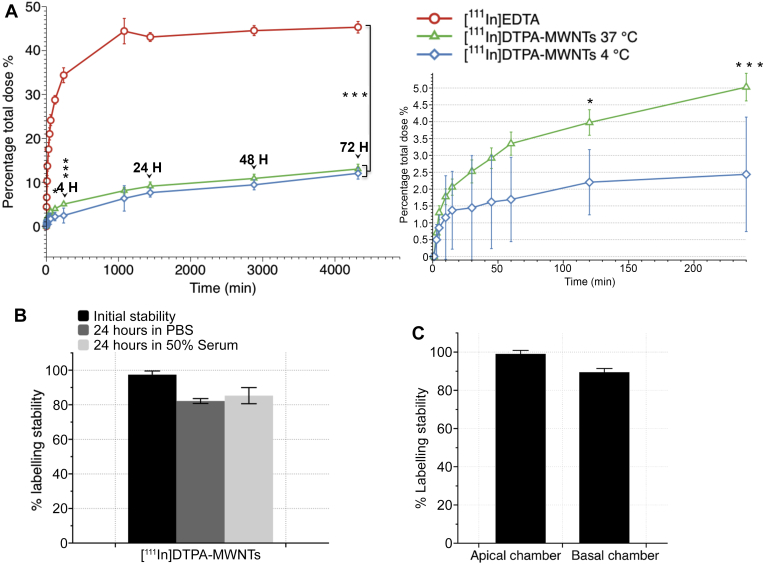
**The percentage of [**^**111**^**In]DTPA-MWNTs transported across the PBEC monolayer over 72 h**. [^111^In]DTPA-MWNTs (20 μg/ml) were added to the apical chamber and incubated with PBEC at 37 °C or 4 °C (for the initial 4 h) followed by 37 °C incubation up to 72 h. The radioactivity in the basal chamber was measured in 0.5 ml aliquots at different time points (**A**). [^111^In]EDTA was used as a control. [^111^In]DTPA-MWNTs incubated at 37 °C crossed the PBEC monolayer and were detected in the basal chamber reaching a maximum of 13.0 ± 1.1% after 72 h. The incubation at 4 °C significantly decreased the uptake of [^111^In]DTPA-MWNTs at 2 h (p < 0.05) and 4 h (p < 0.001) which then increased to the same level as those at 37 °C, reaching a maximum of 12.0 ± 1.5%. The uptake of [^111^In]EDTA showed significantly higher permeation at all time points studied compared to [^111^In]DTPA-MWNTs, reaching a maximum of 45.3 ± 1.3% after 72 h (p < 0.0001). Inset to the right shows the difference in [^111^In]DTPA-MWNTs uptake during the initial 4 h of incubation. (One way ANOVA test; *p < 0.05, ***p < 0.001, ****p < 0.0001, *n* = 3). (**B**) the stability of the radioactive tag on the [^111^In]DTPA-MWNTs as measured by TLC using 0.1 M ammonium acetate buffer containing 50 mM EDTA. Radio-labelled [^111^In]DTPA-MWNTs were incubated with PBS or 50% serum at 37 °C for 24 h prior chromatographic analysis. (**C**) The radiolabelling stability of [^111^In]DTPA-MWNTs during the time course of the transport experiments measured in the apical and basal chamber of the co-culture model, respectively.

The amount of [^111^In]DTPA-MWNTs that crossed the PBEC monolayer into the basal chamber of the Transwell™ system over 72 h incubation period was assessed using ^111^In as radioactive tracer (Fig. 6A). The radioactivity detected in the basal chamber was directly related to the amount of [^111^In]DTPA-MWNTs which crossed the PBEC monolayer. The percentage transport of [^111^In]DTPA-MWNTs was measured at 37 °C and 4 °C. [^111^In]DTPA-MWNTs percentage transport at 37 °C was 5.02 ± 0.4% at 4 h. However, the percentage transported increased considerably after 24 h to 9.1 ± 0.9%, and reached a maximum of 13.0 ± 1.1% at 72 h. Incubating the cells at 4 °C for the initial 4 h resulted in a slight but significantly lower % transport (2.4 ± 0.6%) than that obtained at 37 °C (*p* = 0.0005, One way ANOVA test). This difference was abolished by 72 h upon the re-incubation of Transwells™ at 37 °C (12.0 ± 1.5%).

The stability of the [^111^In]DTPA-MWNTs prior to and during the time course of the study was confirmed using TLC. Fig. 6B shows that [^111^In]DTPA-MWNTs were stable (>80%) after 24 h incubation in PBS or 50% serum at 37 °C making the system suitable to carry out the transport studies. Fig. 6C shows that the measured signals that remained in the apical chamber and the ones transported across PBEC monolayer (sampled from the basal chamber) corresponded to [^111^In]DTPA-MWNTs and not [^111^In]EDTA. This was concluded from the finding that >80% of the signals on the TLC strip remained at the application point (data not shown). This confirms that the radioactivity measured correspond to the transport of labelled [^111^In]DTPA-MWNTs and not [^111^In]EDTA.

To ensure the integrity of the PBEC throughout the transport study and to correct for variations between experiments, [^111^In]EDTA was used as a marker. Fig. 6A shows the transport profile of [^111^In]EDTA with a sharp increase in the transported [^111^In]EDTA over the early time points until it plateaued reaching a maximum of 45.3 ± 1.3% at 72 h. The % transport of [^111^In]EDTA was significantly higher than that of the [^111^In]DTPA-MWNTs at all the time points (*p* < 0.0001), further evidence that signals were related to [^111^In]DTPA-MWNTs transport, and not [^111^In]EDTA. Another permeability marker, [^14^C]sucrose, was used to assess the paracellular permeability in the PBEC BBB model, and to study the potential effect of [^111^In]DTPA-MWNTs on the tight junction stability. Fig. S10 shows that the % permeation of [^14^C]sucrose increased over time and reached a maximum of 35.4 ± 1.5% at 72 h incubation at 37 °C. Incubating the cells with [^14^C]sucrose in the presence of [^111^In]DTPA-MWNTs showed no significant effect on the overall permeation of [^14^C]sucrose into the basal chamber. This was an indirect evidence that [^111^In]DTPA-MWNTs did not damage the cells or the tight junction assemblies, which confirmed the TEM imaging.

Additional experiments were conducted where Transwell™ filters (without PBEC) were used to measure the transport of [^111^In]DTPA-MWNTs, [^111^In]EDTA and [^14^C]sucrose across the filter. [^111^In]DTPA-MWNTs showed higher transport than obtained with PBEC monolayer, and the transport profile was different showing a sharp increase in the measured signal at the early time points in the absence of the PBEC monolayer (Fig. S11). In addition [^111^In]DTPA-MWNTs did not alter the permeation of [^14^C]sucrose across the filter (Fig. S12).

The above studies were all carried out in a serum-free medium to maintain the tight junction assemblies between adjacent endothelial cells. The effect of serum on the [^111^In]DTPA-MWNTs transport across PBEC was assessed in another study comparing the transcytosis rate in the presence or absence of serum. Fig. S13 shows that the transport profile of [^111^In]DTPA-MWNTs across the PBEC monolayer was similar in the presence or absence of serum, but with a slight but significant reduction in transport in the presence of serum.

TEER values were monitored during the time course of the experiment to determine the effect of MWNTs-NH_3_^+^ incubation on the tight junctions between adjacent PBEC. Fig. S14 shows that the incubation of MWNTs-NH_3_^+^ caused TEER to drop from 501.3 ± 183.2 to 219.1 ± 49.2 Ω cm^2^. However, TEER remained above the acceptable >200 Ω cm^2^ threshold throughout the experiment.

The apparent permeability coefficient (P_app_) was calculated for the tested compounds (Table 1). The P_app_ of [^111^In]DTPA-MWNTs was significantly lower than that of [^14^C]sucrose, which indicated that [^111^In]DTPA-MWNTs cross the BBB at a lower rate than sucrose possibly through a different route. As expected, the P_app_ of [^111^In]DTPA-MWNTs at 37 °C was higher than that at 4 °C. In addition, the P_app_ of sucrose was not affected by presence or absence of [^111^In]DTPA-MWNTs.

### 3.8 Bio-distribution of [^111^In]DTPA-MWNTs in mice following i.v. administration

**Fig. 7:**
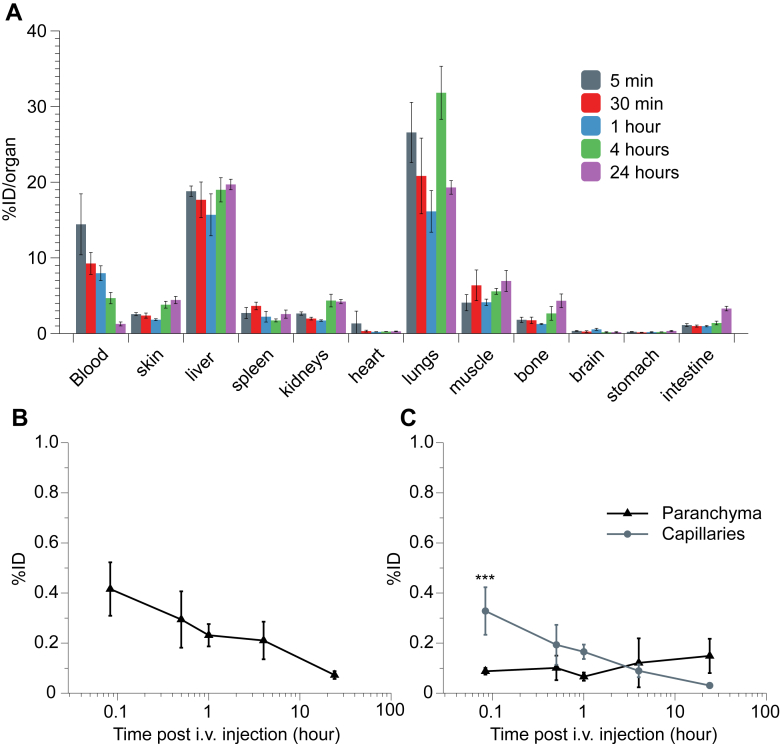
**Organ biodistribution and brain uptake of [**^**111**^**In]DTPA-MWNTs into mice following i.v. administration**. C57/Bl6 mice were injected with [^111^In]DTPA-MWNTs (50 μg, 0.5 MBq) *via* the tail vein. At each time point (5 min, 30 min, 1 h, 4 h and 24 h) whole body perfusion with heparinized saline was performed and major organs were harvested for quantitative measurements of radioactivity by γ-scintigraphy. (**A**) The accumulation of [^111^In]DTPA-MWNTs in the major organs after each time point. (**B**) Brain uptake of [^111^In]DTPA-MWNTs over time showing the highest brain accumulation after 5 min. (**C**) The capillary depletion profile showing the sustained accumulation of [^111^In]DTPA-MWNTs in brain parenchyma over time compared to a gradual reduction in capillary fraction. The data is presented as % injected dose per organ. Results are expressed as mean ± SD, *n* = 3 (***p < 0.001).

The ability of [^111^In]DTPA-MWNTs to accumulate in mouse brain after systemic administration was studied using gamma counting. [^111^In]DTPA-MWNTs was injected in C57/B16 mice *via* i.v. administration and the biodistribution in the major organs was monitored at specific time points. To eliminate interference from blood, animals were perfused with heparinized saline before collecting the organs for gamma counting. Fig. 7A and Fig. S15A shows the biodistribution profile of [^111^In]DTPA-MWNTs in the major organs and the blood. [^111^In]DTPA-MWNTs accumulated mainly in the lungs (26.5 ± 3.9 %ID) and liver (18.8 ± 0.7 %ID) 5 min after injection. The level of [^111^In]DTPA-MWNTs remained relatively high in the lung and liver after 24 h. The blood profile showed that 14.4 ± 0.4 %ID of [^111^In]DTPA-MWNTs were circulating in the blood after 5 min, this level decreased over time to reach 1.3 ± 0.3 %ID after 24 h Fig. 7B shows the level of [^111^In]DTPA-MWNTs accumulation in the brain over time. The highest level was achieved 5 min after injection reaching 0.4 ± 0.1 %ID (≈1.1 ± 0.3 %ID/g). The detected levels of [^111^In]DTPA-MWNTs gradually decreased over time to reach 0.18 ± 0.08% after 24 h.

The capillary depletion method was carried out to examine the distribution of [^111^In]DTPA-MWNTs in the capillaries and brain parenchyma. Fig. 7C shows that a significant amount of [^111^In]DTPA-MWNTs was detected in the capillary fraction (0.3 ± 0.09 %ID) compared to the parenchyma (0.09 ± 0.01 %ID). However, the detected signal in the capillaries decreased over time coupled with a slight increase in the parenchyma fraction. Interestingly, [^111^In]DTPA-MWNTs remained in the parenchyma even 24 h after injection (0.15 ± 0.06 %ID) while the levels in the capillaries diminished considerably (0.03 ± 0.01 %ID). These results provide the first insight into the ability of *f*-MWNTs to reach brain parenchyma following systemic administration.

## 4 Discussion

Despite the promise of amine-functionalized CNT in exerting a therapeutic action in rodent's stroke models, none of the reported studies [4], including ours [5], encountered the blood-brain barrier during the delivery process. CNTs were directly delivered to brain tissues by intra-ventricular [4] or intracranial injection [5]. Only one study administered SWNTs *via* gastro-gavage to deliver acetylcholine (ACh) to the brain of an Alzheimer disease mice model (AD mice). Improvement in learning and memory capabilities were obtained compared to SWNTs alone or to the free ACh. This study provided indirect evidence that SWNTs were able to reach the brain despite the presence of the BBB [25]. However, no direct evidence on brain uptake was provided.

The main focus of the present paper is to examine the ability of MWNTs-NH_3_^+^ to cross PBEC cells *in vitro* and to reach brain parenchyma after systemic injection in mice. MWNTs-NH_3_^+^ were used in this study for the following reasons: (i) they proved their efficacy as siRNA carriers to neural cells after local injection in the brain [5] (ii) they elicited no inflammatory response compared to the oxidised equivalent, ox-MWNTs-NH_3_^+^, which caused a reversible transient immune response [26]; (iii) biodegradation of MWNTs-NH_3_^+^ in brain cells, following stereotactic injection in the brain cortex of mice, has been shown microscopically [27]. Moreover, we hypothesize that the slightly cationic nature of the MWNTs-NH_3_^+^ could improve their cellular uptake into endothelial cells *via* adsorptive-mediated endocytosis routes [28].

The main obstacle in studying the mechanism of nanoparticle uptake across the BBB is finding a representative *in vitro* BBB model which closely mimics the characteristics of the *in vivo* BBB. Several models have been described using immortalized endothelial cell lines such as mouse brain endothelial cells bEnd.3 [29], rat brain endothelial cells RBE4 [30,31], and hCMEC/D3 human endothelial cells [32]. Primary cultures of brain endothelial cells provide a better polarized BBB model compared to immortalized cell lines. Some examples include primary bovine brain microvessels endothelial cells (BMEC) [33] and porcine brain endothelial cells [34]. Evidence from a variety of studies confirms that co-culture of primary endothelial culture with primary astrocytes gives more complete BBB-like features by increasing the BBB phenotype and reducing non-brain endothelial specific features. The quality of the BBB model is generally assessed by TEER measurement, and permeability index [11].

The PBEC/astrocyte BBB model used in this experiment is a well-established model that has been previously reported and characterized. Rubin et al. described the isolation of bovine brain endothelial cells and the successful establishment of an *in vitro* co-culture model of endothelial cells and primary astrocytes [10]. Based on this model, PBEC have been isolated with modifications by Skinner et al. and were used to set up a BBB model of PBEC and primary astrocytes identical to that used in our work [19]. TEER was used to characterize the tightness of the PBEC monolayer, which reflected the state of tight junctions between the cells. Even though no cell surface markers were used to characterise PBEC, the high TEER values were characteristic of brain capillary endothelial cells. This is confirmed by the work of Butt et al. who showed the difference between TEER in brain surface (BBB) pial microvessel (>1000 Ω cm^2^) compared to TEER reported in the peripheral microvessels (2–20 Ω cm^2^) [35]. Therefore, in our model, we relied on TEER measurement to assess the presence of tight junctions indicative of healthy brain endothelial cells. TEER >200 Ω cm^2^ was the threshold below which we rejected the model.

A key advantage of using a co-culture model is enhancing the polarity of the PBEC monolayer and inducing the expression of surface receptors that distinguish brain endothelial cells from peripheral endothelial cells [36]. The polarity of the endothelial cells is vital when studying transcytosis across the BBB and for the expression of luminal/abluminal-specific receptors and transporters. Laakkonen et al. have studied the transcytosis of adenovirus vectors across hCMEC/D3 cells, and showed a marked difference in the transduction between polarized hCMEC/D3 grown on Transwell™ filters coated with rat-tail collagen type I and the un-polarized hCMEC/D3 cells grown in cell culture plates [36].

Co-culture models have been used to study nanoparticle transport across the BBB. The uptake of transferrin-decorated liposomes [37], citrate-coated gold nanospheres [38] and gold nanoparticles [39] was investigated in the hCMEC/D3 *in vitro* BBB model [37–39]. Superparamagnetic iron oxide and polymeric nanoparticle uptake across the BBB was studied in a co-culture model of human brain capillary endothelial cells obtained from a female epilepsy-sufferer and immortalized by transfection with simian virus 40 T antigen [40,41]. Silver nanoparticles were applied in a co-culture of rat brain microvessel vascular endothelial cells BMVECs [42]. However, to our knowledge no report has been published on the uptake characteristics of CNTs in any BBB model *in vitro* or *in vivo*. The closest was a study by Bhattacharya et al. who examined the uptake of SWNTs-DNA hybrids into human umbilical vein endothelial cells (HUVECs). The SWNTs-DNA constructs were able to enter the cells and to accumulate within vesicles after 6 h of incubation [43]. The mechanistic studies carried out using specific endocytosis inhibitors pinpointed macropinocytosis as the main route of uptake as opposed to clathrin- or caveolae-dependant endocytosis. In the present study, the behaviour of MWNTs-NH_3_^+^ in PBEC was similar to that of SWNTs-DNA in HUVECs. MWNTs-NH_3_^+^ were able to enter and accumulate within endocytic vesicles within 24 h followed by complete transcytosis for some of the vesicles. Incubating the cells at 4 °C significantly reduced the uptake and transcytosis as shown by the [^111^In]DTPA-MWNTs transport study. This was supported by the STEM images showing the MWNTs-NH_3_^+^ interacting with PBECs, but not within the cell at 4 °C. These observations confirmed the involvement of an energy-dependant process in the transcytosis of MWNTs-NH_3_^+^ across the PBEC layer. Morphological observations by STEM suggested that macropinocytosis is the main route of uptake mainly due to the size of the MWNTs-NH_3_^+^ and the observation of lipid ruffles engulfing the MWNTs-NH_3_^+^, which is characteristic of macropinocytosis [44]. However, more studies are needed to confirm the specific endocytic route(s) involved in MWNTs-NH_3_^+^ uptake across PBEC.

Studying the uptake of CNTs into endothelial cells has been previously reported using light microscopy [45] and conventional TEM [46]. The use of low voltage STEM imaging to study the uptake and transcytosis of MWNTs-NH_3_^+^ in PBEC provided several advantages as we show for the first time the transport of MWNTs-NH_3_^+^ over 48 h in three distinct detection modes. The ability to image the cells using BF, ADF and HAADF in parallel provided an invaluable tool to analyze the interaction between MWNTs-NH_3_^+^ and the osmium-stained cellular membranes e.g. plasma membranes and endocytic membranes. Previous studies have explored high-end imaging tools to study the interaction of CNTs with immortalized cells and primary cultures [7,47], but none has used a fully polarized cell type as in this BBB model, which is important in reproducing the vectorial movement of nanoparticles.

Only two earlier studies report the use of PBEC to study the uptake of nanoparticles. PBEC were used to study the uptake characteristics of third-generation (G3) PAMAM nanoparticles conjugated to paclitaxel. The data showed that conjugating the drug molecule to PAMAM resulted in significantly higher transport rate across the PBEC monolayer compared to the drug alone [48]. Freese et al. have investigated the transcellular transport of gold nanoparticles across a PBEC/astrocytes co-culture model using TEM. The results showed that gold nanoparticles were able to enter the cells and accumulate in vesicles within 24 h. When the experiment was carried out with hCMEC/D3 cells the vesicles containing the gold nanoparticles were observed in the perinuclear region. However, the fate of the gold nanoparticles entering the endothelial cells was not discussed in either model [39]. In our experiments, the MWNTs-NH_3_^+^ accumulated in multi-vesicular bodies within 24 h, but MWNTs-NH_3_^+^-containing vesicles were observed in the para-junctional zones rather than the perinuclear region. The data presented here showed the complete crossing of MWNTs-NH_3_^+^ in the PBEC layer after 72 h. Therefore, we were able to demonstrate, for the first time, the ability of MWNTs-NH_3_^+^ to cross the BBB *in vitro* with low voltage STEM imaging, thus providing solid evidence using electron microscopy for each step of the transcytosis process. Crossing the PBEC monolayer by transcytosis was also confirmed by tracking the MWNTs-NH_3_^+^ into the primary rat astrocytes. TEM images of the astrocytes revealed the presence of MWNTs-NH_3_^+^ that successfully crossed the PBEC monolayer. This observation consolidated the conclusion regarding the crossing of the BBB *in vitro* by MWNTs-NH_3_^+^. However, the route of uptake into the astrocytes was different from that in PBEC. MWNTs-NH_3_^+^ appeared as individual particles as opposed to the clusters within PBEC. This pattern of nanoparticle uptake into astrocytes was previously reported in a study that tracked the uptake of glucose-coated gold nanoparticles across a 3D co-culture of hCMEC/D3 and primary human astrocytes. Similar to our findings, the gold nanoparticles were found within the astrocytes after 8 h of incubation with the endothelial cells with no evidence of vesicular uptake [49].

“Endosomal escape” was observed in a few images. However, lone MWNTs-NH_3_^+^ were not detected within the PBEC cytosol, as ADF and HAADF images always identified a very thin membrane surrounding the individual MWNTs-NH_3_^+^ within the cytoplasm. Moreover, MWNTs-NH_3_^+^ were not observed in either the nucleus or the mitochondria at any time point; this is in contrast to the reported accumulation of CNTs in the nucleus and mitochondria of certain cell preparations, for example the lung epithelial cell line A549 [46,50]. The uptake experiments using [^111^In]DTPA-MWNTs showed that despite incubation at 4 °C, a small percentage of [^111^In]DTPA-MWNTs were transported into the basal chamber. Moreover, [^111^In]DTPA-MWNTs transport across PBEC showed lower P_app_ than [^14^C]sucrose indicating that [^111^In]DTPA-MWNTs crossed the BBB at a lower rate which is expected for the transcellular uptake route. The slow transport rate observed during the initial 4 h of incubation (by radiolabelling) agreed well with TEM/STEM images, which showed crossing of the MWNTs-NH_3_^+^ only after 24 h. The fact that P_app_ of [^111^In]DTPA-MWNTs at 37 °C was higher than that at 4 °C, further confirmed the involvement of energy-dependant uptake of [^111^In]DTPA-MWNTs across the BBB.

The mechanistic *in vitro* studies showed extensive evidence of the inherent ability of MWNTs-NH_3_^+^ to cross the BBB. The organ biodistribution profile suggested brain accumulation of [^111^In]DTPA-MWNTs into mouse brain following intravenous administration. However, the majority of [^111^In]DTPA-MWNTs was found in the liver and lungs. This was not surprising as the same observation was made in our previous study [51]. The highest whole brain accumulation was obtained at 5 min post-injection (1.1 ± 0.3 %ID/g). This value may seem modest compared to other organs, but it is within the range of values reported for other types of nanoparticles, which have shown success in brain delivery [52,53]. It is emphasized that whole body saline perfusion was carried out before harvesting organs so that interference with radioactivity in circulating blood is eliminated.

Previously liposomes have been used to deliver the BBB impermeable drug serotonin to mice brain using i.v. administration. The radiolabelled non-PEGylated liposomal serotonin achieved higher brain accumulation compared to free serotonin after 1 and 24 h. The same study reported whole brain uptake of 0.138 ± 0.034 and 0.097% ± 0.011 %ID/g of brain after 1 and 24 h, respectively [52]. Interestingly, the non-PEGylated [^111^In]DTPA-MWNTs reported in this study achieved 8-fold higher whole brain uptake than the liposomes reported by Afergan et al. [52]. Another study using radiolabelled passively targeted PEGylated liposomes reported whole brain uptake of 0.69 ± 0.06% %ID/g of after 1 h of administration. This value is also significantly lower than values we report here.

In addition to significant brain uptake reported for [^111^In]DTPA-MWNTs, the brain uptake is much faster than that reported for other nanocarriers. Notably, the highest value (C_max_) of [^111^In]DTPA-MWNTs in brain was achieved at 5 min post-injection. C_max_ of liposomes, for example, was obtained at a later time points 1 h post-injection [52,53]. Capillary depletion results indicate rapid interaction with brain endothelium (∼5 min), which may have contributed to the highest whole brain uptake value, at the early time point. No time points earlier than 5 min were examined in this study. Interestingly, the concentration in brain parenchyma was found to be sustained over the 24 h period, which suggest either a continuing entry into the brain parenchyma or the limited clearance over the studied period.

NPs have also been studied for brain targeting using surface ligands. Lockman et al., studied the brain accumulation of solid NPs made from oil-in-water microemulsions after coating with thiamine to target the thiamine receptors on the brain endothelium. The data showed that 0.5 %ID (≈1.1 %ID per gram of tissue) of the injected NPs-thiamine accumulated in the brain 2 h after injection [54]. Another study used lactoferrin-conjugated liposomes to investigate brain targeting in mice following i.v. administration. Lactoferrin-conjugated liposomes reached 1.02 ± 0.06 and 0.81 ± 0.05 %ID/g in mouse brain after 2 and 6 h, respectively [53]. Coincidentally, both studies reported similar values to our biodistribution study even though the [^111^In]DTPA-MWNTs were not actively targeted to the BBB. This highlights the importance of the data we present here which is likely to improve when BBB targeting ligand is conjugated to *f*-MWNTs.

## 5 Conclusions

This was the first evidence for the unique ability of *f*-MWNTs to cross the *in vitro* co-culture BBB model and to accumulate in mouse brain following systemic administration *in vivo*. The choice of the co-culture BBB model was based on the superiority of co-cultured primary endothelial cell cultures in forming tight junctions, expressing surface receptors, and demonstrating transcytosis, thus mimicking the *in vivo* BBB characteristics more closely than in immortalized endothelial cell lines. Ultra-structural imaging by TEM and STEM was employed to gain unequivocal evidence for translocation of the intact MWNT-NH_3_^+^, which also confirmed the route(s) of uptake. Electron micrographs confirmed transcytosis of MWNT-NH_3_^+^ and its sequence as a function of time. MWNT-NH_3_^+^ were found within endocytic vesicles and multi-vesicular bodies within 4–24 h of incubation, followed by a basal exit from PBEC cells after 48 h. ADF and HAADF images showed clearly the lipid membranes surrounding the vesicles and the presence of intact plasma membranes following MWNT-NH_3_^+^ exit, which in combination with the modified LDH assay indicated the biocompatibility of MWNT-NH_3_^+^ vectors. Most importantly, the presence of *f*-MWNT in the astrocyte layer was also confirmed by TEM. The extent of *f*-MWNTs transport across the PBEC monolayer was investigated by tracking the radiolabelled [^111^In]DTPA-MWNTs from apical to basal chamber. This is the first evidence of *f*-MWNT translocation across the BBB in an *in vitro* model. The significant reduction in the transport of *f*-MWNT across the BBB at 4 °C confirmed that the uptake was driven by an energy-dependent pathway. *In vivo* bio-distribution studies showed that a substantial amount of *f*-MWNTs accumulated at early time points in mouse brain following systemic administration. Capillary depletion provided initial evidence on the presence of *f*-MWNTs within brain parenchyma. Future work will focus on surface modification of *f*-MWNTs, of different diameters, with BBB targeting ligands to examine whether brain uptake can be further enhanced.

## Figures and Tables

**Table 1:** Apparent permeability coefficient (Papp) of [^111^In]DTPA-MWNTs and [^14^C]sucrose across PBEC.

	[^111^In] MWNTs (37 °C)	[^111^In] MWNTs (4 °C)	[^111^In]EDTA (37 °C)	[^14^C]sucrose (37 °C)^a^	[^14^C]sucrose (37 °C)^b^
P_app_ (cm/s) × 10^−6^	0.2 ± 0.02	0.05 ± 0.02	1.03 ± 0.06	8.6 ± 2.1	8.9 ± 0.8

Values are presented as mean ± SD, *n* = 3.

a In the absence of [^111^In] MWNTs.

b In the presence of [^111^In] MWNTs.
