# Supplementary material for: The interaction of carbon nanotubes with an in vitro blood-brain barrier model and mouse brain in vivo
Source: Biomaterials. 2015 Jun;53:437–52. doi: 10.1016/j.biomaterials.2015.02.083 (PMC4407899; doi:10.1016/j.biomaterials.2015.02.083)
Supplement: Supplementary file 1 [file mmc1.docx]

**The interaction of carbon nano-needles with an in vitro blood brain barrier model and mice brain in vivo**

Houmam Kafa^1^, Julie Tzu-Wen Wang^1^, Noelia Rubio^1^, Kerrie Venner^2^, Glenn Anderson^3^, Elzbieta Pach^4^, Belén Ballesteros^4^, Jane Preston^1^, N Joan Abbott^1^ and Khuloud T Al-Jamal^1^*

^1^Institute of Pharmaceutical Science, King's College London, Franklin-Wilkins Building, 150 Stamford Street, London SE1 9NH (UK)

^2^Institute of Neurology, University College London, Queen Square, London WC1N 3BG (UK)

^3^Histopathology Department, Great Ormond Street Hospital for Children, London WC1N 3JH (UK)

^4^ICN2 - Institut de Catala de Nanociencia I Nanotecnologia, Campus UAB, 08193 Bellaterra, Barcelona (Spain)

Address correspondence to:

* Dr Khuloud T. Al-Jamal

Institute of Pharmaceutical Science

King's College London

Franklin-Wilkins Building

150 Stamford Street

London SE1 9NH, UK

E-mail: [khuloud.al-jamal@kcl.ac.uk](mailto:khuloud.al-jamal@kcl.ac.uk)

SI TEXT

**Supplementary Methods**

## *Functionalization of pristine multi-walled carbon nanotubes by 1, 3-dipolar cycloaddition reaction*

Pristine MWNTs (20 mg), with a diameter of 20-30 nm, were suspended in 20 ml of dimethylformamide (DMF) and sonicated for 10 min. After sonication, the tertiary-butyloxycarbonyl (Boc)-protected amino acid (150 mg, 0.51 mmol) and paraformaldehyde (18.5 mg, 0. 51 mmol) were added stepwise (30 mg amino acid and 3.7 mg aldehyde every 24 h), and the mixture was heated at 125 ^◦^C for 5 days. Multiple centrifugation steps (1811 g, 10 min) were carried out to separate the unreacted MWNTs (remain suspended) from the *f*-MWNTs, which were then filtered through a 0.2 μm PTFE filter and the collected black solid was washed with 100 ml of dimethylformamide (DMF) and methanol yielding 18 mg of *f*-MWNTs.

To eliminate the Boc protecting group, *f*-MWNTs (15 mg) were suspended in 10 ml DMF, and sonicated for 10 min. After sonication, 10 ml of trifluoroacetic acid (TFA) was added to the reaction mixture. The solution was stirred for 24 hours and the *f*-MWNTs were filtered through a 0.2 μm PTFE filter. The collected black solid was washed with 10 ml of DMF and methanol yielding 13 mg of amino-functionalized MWNTs (MWNTs-NH_3_^+^). The synthesized MWNTs were characterised by [Thermogravimetric analysis](http://en.wikipedia.org/wiki/Thermogravimetric_analysis) (TGA) and Kaiser test. The synthesis of Boc- protected amino acid is described in [1, 2].

## *Synthesis of DTPA-MWNTs*

MWNTs were dispersed in 10 ml of dry DMF, and the ammonium groups were neutralised with diisopropylethylamine (2.2 µl, 15.87 µmol at 3:1 excess molar ratio). Diethylene triamine pentaacetic acid (DTPA) was added to the MWNTs (18.89 mg, 52.9 µmol at 10:1 excess molar ratio), and the reaction was stirred for 48 hours at 60 ^◦^C under nitrogen atmosphere to avoid the hydrolysis of the anhydride groups in the presence of water in air. The mixture was filtered through a 0.2 μm PTFE filter, re-suspended in 100 ml DMF and sonicated for 10 min. Several washing steps were then carried out on the sample in 1:1 methanol water solution to remove unbound DTPA from the mixture. The filtrate was re-suspended in 10 ml of methanol and dialysed against deionised water (MW cut off size: 10,000 Dalton) for 48 hours at 4 ^◦^C in water. Finally, the mixture was filtered and re-suspended in water to a concentration of 1 mg/ml. The synthesized MWNTs were characterised by TGA and Kaiser Test.

***Kaiser Test***

Ninhydrin assay reagents (phenol/ethanol 42.55 M, KCN in pyridine 0.02 M and Ninhydrin/ethanol 0.28 M) were added to 0.27 mg of MWNTs-NH_3_^+^ and DTPA-MWNTs. The two suspensions were sonicated for 1 min and then heated at 100C for 7 min followed by the addition of 4.5 ml ethanol:water (60:40). The suspensions were centrifuged at 1811 g for 10 min. The UV-vis absorbance was measured at λ=575 nm in Lambda 35 spectrophotometer (Perkin Elmer, USA).

### [*Thermogravimetric analysis*](http://en.wikipedia.org/wiki/Thermogravimetric_analysis) *(TGA)*

Samples of pristine MWNTs, MWNTs-NH_3_^+^ and DTPA-MWNTs were loaded in platinum pans and the TGA analysis was performed using the TGA Q500 (TA instruments, USA). Initially the analysis was carried out isothermally in nitrogen atmosphere at 100 ^◦^C for 20 min followed by a controlled increase in temperature at a rate of 10 ^◦^C/min to reach a maximum of 1000 ^◦^C at the end of the analysis.

**Supplementary Results**

***The effect of surface charge of f-MWNTs on crossing the BBB in vitro***

To examine the effect of *f*-MWNTs surface charge on crossing the BBB, further TEM imaging was employed to study the uptake of DTPA-MWNTs and MWNTs-COOH. The former carries a slight positive charge whereas MWNTs-COOH are negatively charged. Both derivatives were incubated with the co-culture model, and the uptake was studied with TEM. **Figure S6** shows that both derivatives interacted with PBEC triggering the uptake *via* vesicular route identical to that observed with MWNTs-NH_3_^+^. The images confirm that crossing of the BBB *in vitro* is independent of the surface charge, and is a function of the *f*-MWNTs. Further systematic studies concerning the effect of surface charge on the uptake will be carried out in the future to examine the consequences of *f*-MWNTs surface charge on uptake kinetics.

## *Stability of MWNTs-NH_3_^+^ within the cells following uptake into PBEC*

In addition to the importance of stability of the loaded therapeutic cargo, it was important to assess the stability of the MWNTs-NH_3_^+^ within the endothelial cells, which is vital from a drug delivery point of view where the MWNTs-NH_3_^+^ should cross the BBB without losing their structure. HRTEM and EELS were used to study the fine structure of MWNTs-NH_3_^+^ after uptake into PBEC, which should reflect their stability in the cells. **Figure S7 A** shows high-resolution images of MWNTs-NH_3_^+^ after uptake. The multi-walled structure of the MWNTs-NH_3_^+^ was clearly visible in the inset images and the interlayer spacing between the walls of the MWNTs was found to be 0.34 nm, indicating that the graphitic structure of the MWNTs-NH_3_^+^ was not altered within the endothelial cells.

EELS analysis relies on measuring the kinetic energy of the electrons after hitting the sample. Different elements within the sample cause a reduction in the energy of the electron as it hits the sample. This loss of energy can be recorded with an electron spectrometer, and is directly related to the nature of the elements within the sample. Therefore, EELS presents an invaluable tool for elemental analysis of samples. STEM-EELS analysis was used in our experiments to study the fine structure of MWNTs-NH_3_^+^ **(Figure S7 B)**. The spectra of the MWNTs-NH_3_^+^ within and outside the cells were compared to the background signals obtained from the endothelial cells, the polyester filter (support material used to grow the cells on) and the resin used for the TEM sample preparation. In all cases a carbon π* band transition was observed at 285 eV, with higher intensity for MWNTs-NH_3_^+^ areas **(Figure S7 B, points 1** and **2)**. The relative intensity of this peak is a measure of the sp^2^ predominance over sp^3^ hybridization, a high sp^2^ content being characteristic of graphitic structures such as that of MWNTs-NH_3_^+^. On the other hand, the σ* peak at ~292 eV, characteristic of graphitic material of the MWNTs-NH_3_^+^, was detected in MWNTs-NH_3_^+^ points (points 1 and 2), but was not present at the background areas (points 3, 4 and 5). The EELS spectra acquired from the cells, polyester filter or the resin show absence of these features as these are formed mainly of sp^3^ carbon. To summarize, HRTEM and STEM-EELS both confirmed the integrity of the graphitic structure of the MWNTs-NH_3_^+^ following uptake into PBEC. This result agrees well with the radiolabelling stability assessment after crossing the PBEC/filter layer.

The stability of nanoparticles is of a great importance in biological applications and CNTs show high structural stability in the biological environment, which raises some concerns from a toxicological point of view. We previously reported the biodegradation of MWNTs-NH_3_^+^ in mouse cortex two days after intracranial injection [3]. In the present study we examined the stability of the MWNTs-NH_3_^+^ *in vitro* using EELS and showed no alteration in the graphitic structure of the MWNTs-NH_3_^+^ after 48 hours of incubation with PBEC. Moreover, the structural integrity of the MWNTs-NH_3_^+^ was retained even when the ultrathin sections were subjected to high acceleration voltage of 120 kV, which exceeds the 80 kV threshold above which knock-on damage is of concern for carbon-based samples [4]. Further studies are required to examine the trafficking of MWNTs-NH_3_^+^ across the *in vivo* BBB, their stability in the brain microenvironment, their pharmacokinetics in the brain and mechanism of clearance.

## *Transport of radiolabeled MWNTs-DTPA,* [^111^In]EDTA and [^14^C]sucrose *across the polycarbonate filter in the absence of PBEC monolayer.*

Due to the porous nature of the Transwell™ filter, we expected the filter to adversely affect [^111^In]DTPA-MWNTs transport into the basal chamber. Therefore, additional controls of Transwell™ filters (without PBEC) were used to measure the transport of [^111^In]DTPA-MWNTs across the filter to estimate the maximum possible transport that could be obtained. The transport profile of [^111^In]DTPA-MWNTs in the absence of PBEC showed a sharp increase in the early time points in contrast to the slower transport observed in the incubation with PBEC (**Figure S11)**, reaching a maximum of 19.4 ± 1.1 % after 72 hours. [^111^In]EDTA, as a small molecule, showed a sharp increase in the % transport in the absence of PBEC during the initial 5 min (28.7 ± 0.9 %), then further increased over time to reach a plateau at 72 hours (42.3 ± 0.8 %). The co-incubation of [^111^In]DTPA-MWNTs and [^14^C]sucrose **(Figure S12)** resulted in no significant change in [^14^C]sucrose transport across the filter, as seen with cells. This showed that although aggregates of [^111^In]DTPA-MWNTs are prone to being trapped in the filter pores, they did not completely block the pores, thus allowing permeation of [^14^C]sucrose across the filter to proceed.

***The effect of MWNTs-NH­_3_^+^ exposure on TEER***

Trans-endothelial electrical resistance (TEER) was used to assess the tight junction formation and therefore the integrity of the PBEC monolayer at the start of each experiment. TEER > 200 Ω.cm^2^ was used as a threshold to accept or reject the co-culture before introducing the MWNTs-NH­_3_^+^ onto the layer. The TEM images showed that it was not feasible for the MWNTs-NH­_3_^+^ to cross the PBEC monolayer through the tight junctions due to the length of the MWNTs-NH­_3_^+^ (Median length = 500 nm). Therefore, TEER > 200 Ω.cm^2^ was deemed acceptable in the present experiments.

We have also examined the effect of MWNTs-NH­_3_^+^ on TEER for up to 72 hours which is the maximum experimental duration used. **Figure S14** below shows the TEER of the PBEC monolayer after introducing the MWNTs-NH­_3_^+^. Cells incubated with [^14^C]sucrose or [^111^In]EDTA were used as controls. Incubating the cells with MWNTs-NH­_3_^+^ caused a drop in TEER from 501.3 ± 183.2 to 219.1 ± 49.2 Ω.cm^2^ at 24 hours. However, TEER values remained higher than 200 Ω.cm^2^ indicating the presence of intact tight junctions. TEER in control groups were higher than MWNTs-NH­_3_^+^ treated groups at all time points. In this experiment, it was not possible to evaluate if the drop in TEER was reversible as the PBEC were fixed for TEM imaging at the end of each time point. Furthermore, in a study by Cellot *et al.* Carbon nanotubes (CNTs) showed improved neuronal responsiveness by creating electrical shortcuts between the cells due to their inherent electronic properties [5]. It is possible that the drop in TEER in this study was due to the effect of CNTs on electron’s flow from the apical to the basal chamber resulting in lower TEER values. This hypothesis is supported by TEM images of PBEC showing intact tight junctions with no evidence of MWNTs-NH­_3_^+^ disrupting the layer or tight junctions.


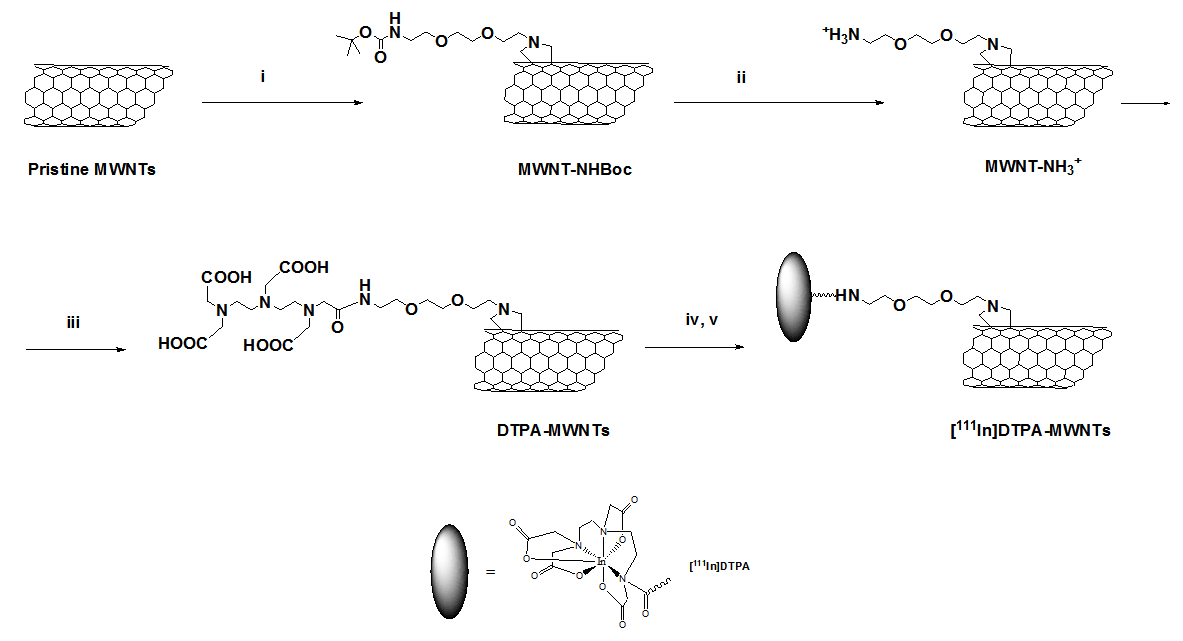


***Scheme S1: Synthesis of MWNTs derivatives.*** **(i)** Boc protected aminoacid (CH_3_)OCONH(CH_2_CH_2_O)_2-_CH_2_CH_2_NHCH_2_COOH, HCHO, in DMF, 125C, 5 days. **(ii)** CF_3_COOH in CH_2_Cl_2_, stirring, 24 h. **(iii)** DTPA, Et_3_N in DMF, 60C, 48 h, N_2_ atmosphere. **(iv)** ^111^InCl_3,_ at room temperature in 0.1 M ammonium acetate buffer (CH_3_COONH_4_) pH 5.5 for 30 min.


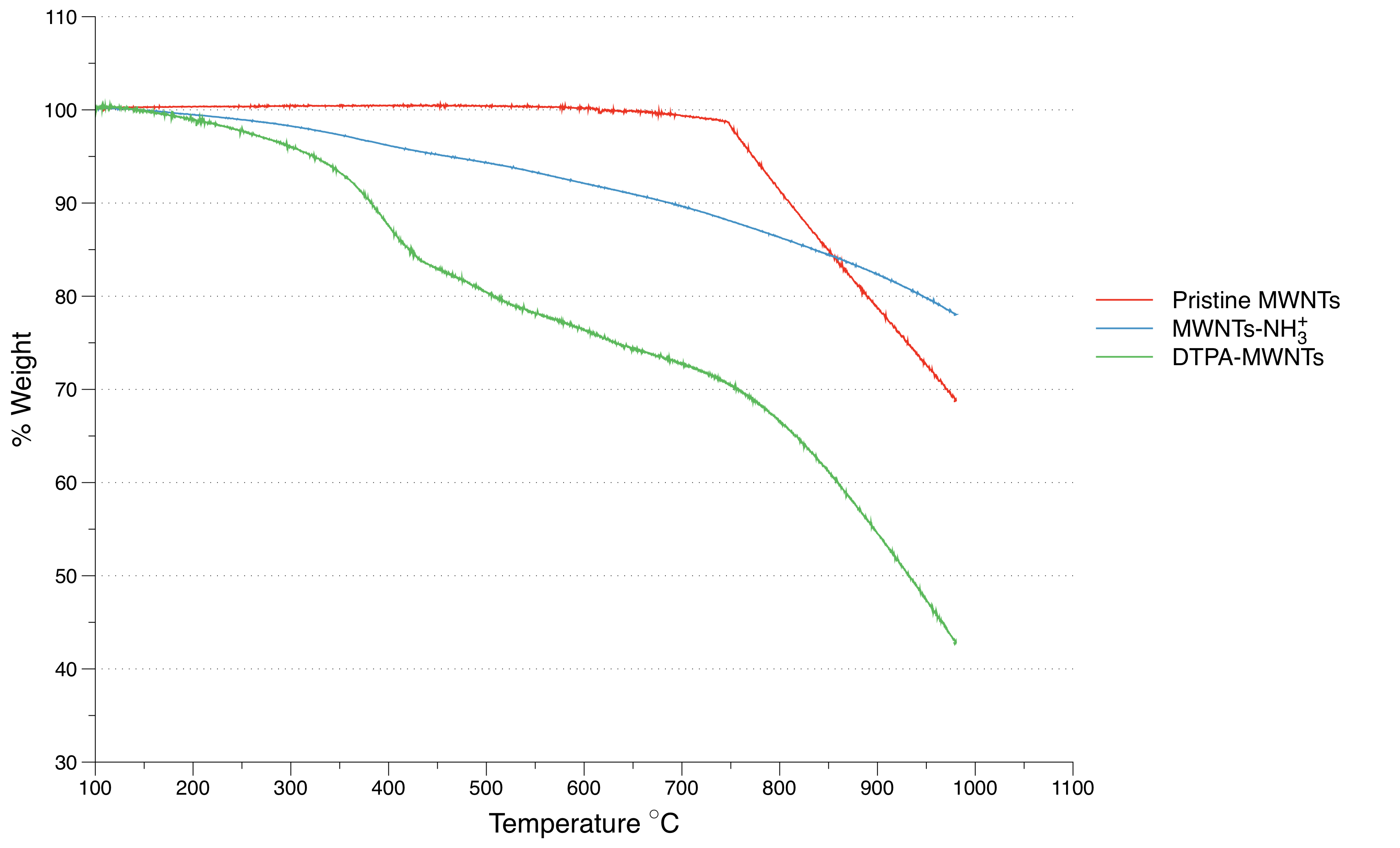


**Figure S1: Thermogravimetric analysis of pristine MWNTs, MWNTs-NH_3_^+^ and DTPA-MWNTs.** The percentage mass loss is the result of the degradation of the organic functional groups present on the side walls of the MWNTs. The pristine material was stable up to 750^◦^C above which it started to decompose. MWNTs-NH_3_^+^ showed mass loss (9 % at 600 ^◦^C corresponds to 529 µmol/g of MWNTs) indicating the addition of functional groups to the structure. Also the degradation of the MWNT-NH_3_^+^ started at a lower temperature (approx 750^◦^C) compared to pristine MWNTs. The DTPA-MWNTs showed the highest mass loss (26 % at 600 ^◦^C corresponds to 493 µmol/g of MWNTs which combined with negative results obtained with Kaiser test confirmed the coupling of DTPA molecules to the functional amine groups. The µmol of functional groups was calculated from the percentage mass loss and the molecular weight of the side chains.


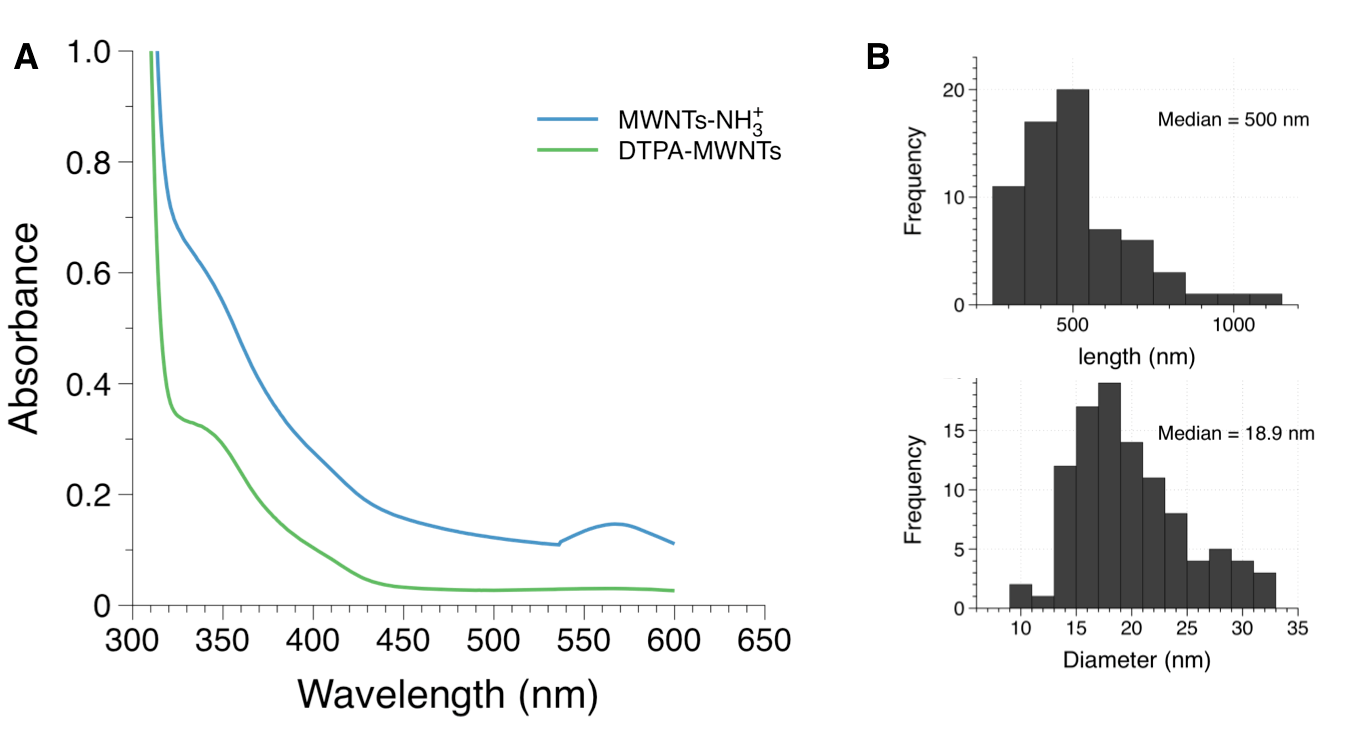


**Figure S2: UV-vis absorbance of the synthesized MWNTs-NH_3_^+^ and DTPA-MWNTs.** UV-vis spectra **(A)** showing the outcome of Kaiser Test reaction. The absorbance at λ=575 nm (0.14, corresponds to 120.6 µmol/g of MWNTs-NH_3_^+^) indicates the generation of the primary amine groups after 1,3-dipolar cycloaddition reaction/ de-protection which were available to react with DTPA. The UV-vis absorbance of the DTPA-MWNTs shows a flat peak at λ=575 nm (0.03, corresponds to 25.8 µmol/g of DTPA-MWNTs) indicating the conversion of primary amines into DTPA conjugates. **(B)** Length and diameter distribution histogram (*n* = 100) of the studied MWNTs-NH_3_^+^. The median length and diameter of the MWNTs-NH_3_^+^ was 500 nm and 18.9 nm, respectively.


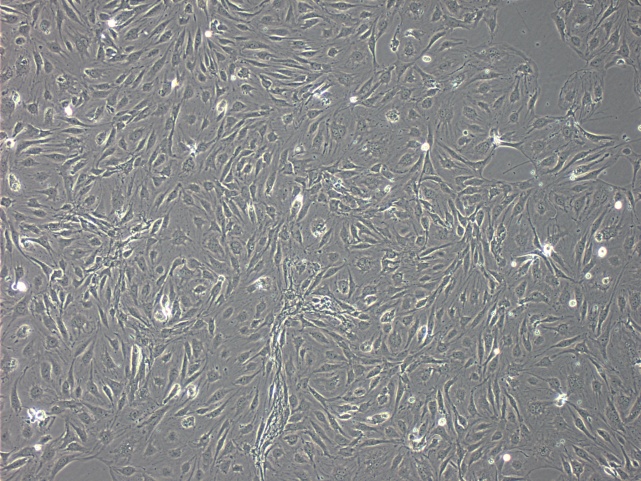

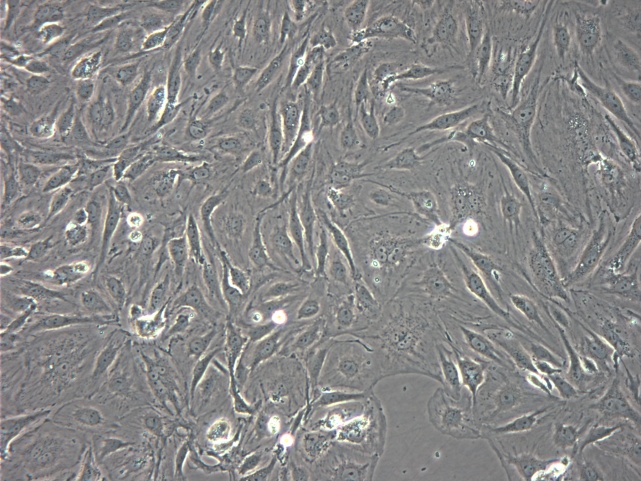

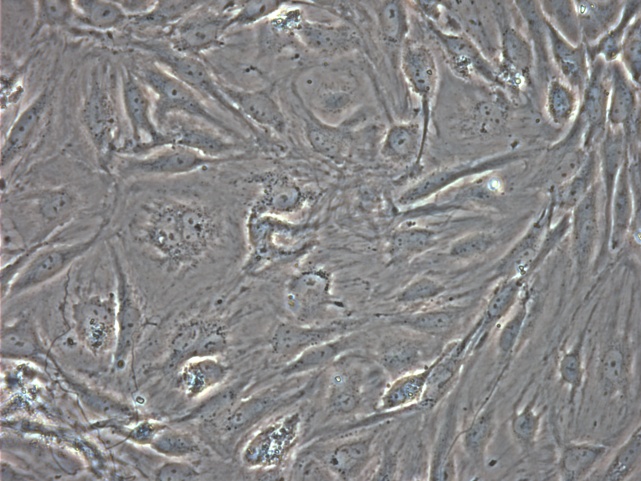


**Control**

**20 µg/ml**


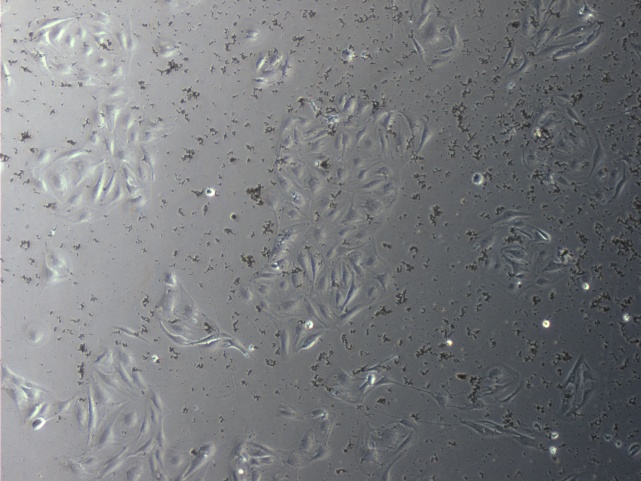

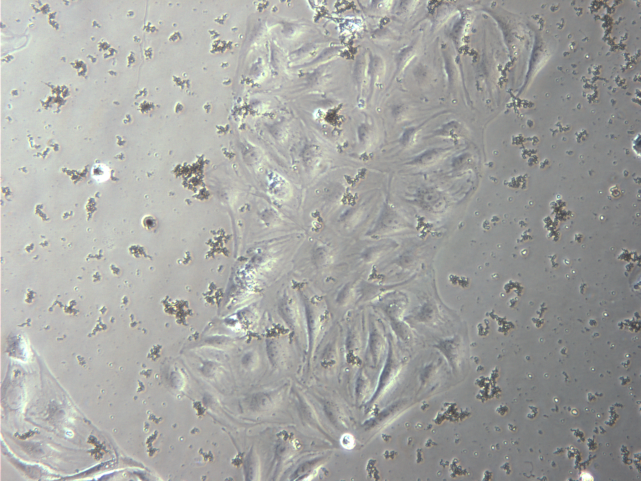

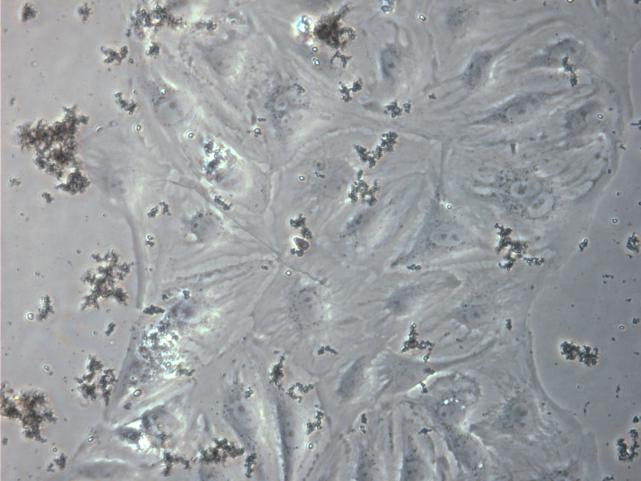


**50 µg/ml**


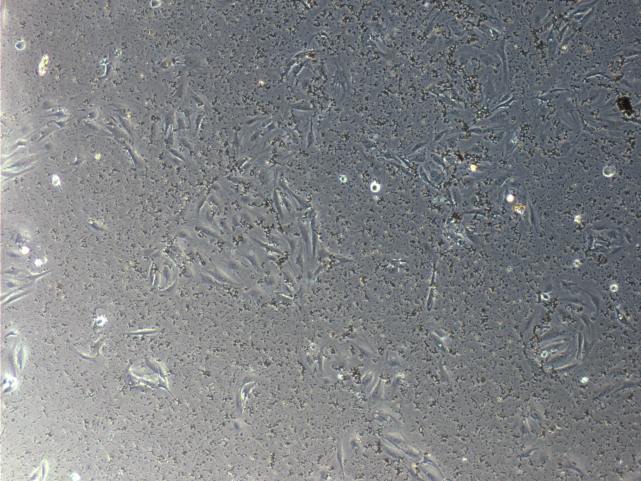

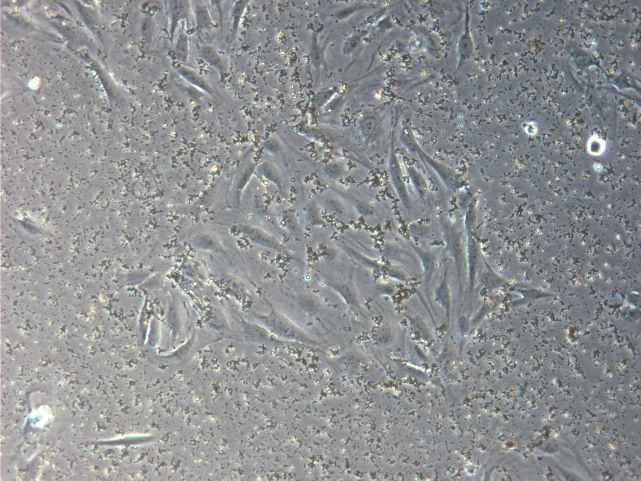

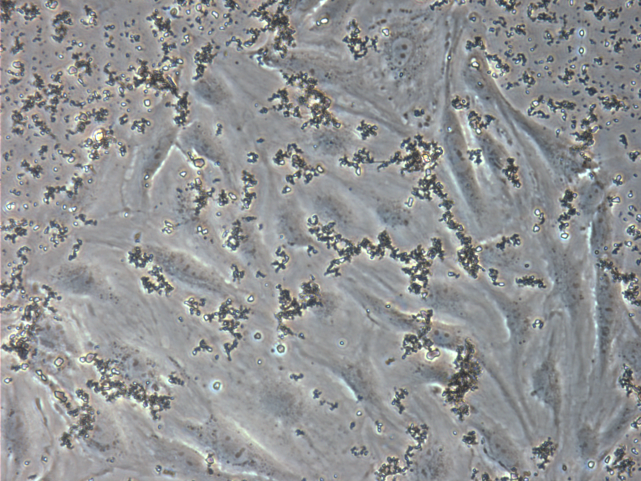

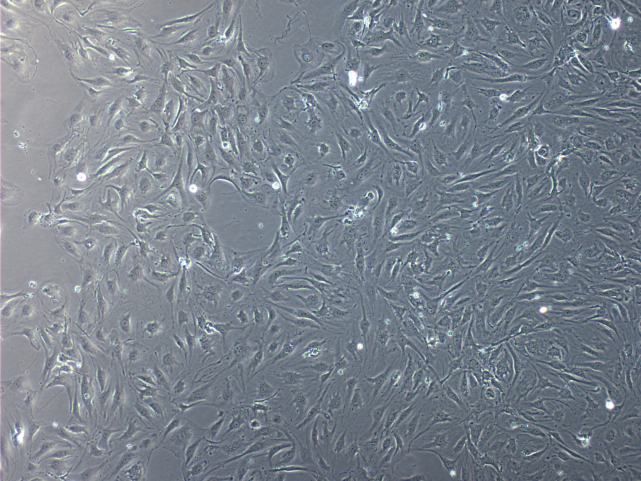

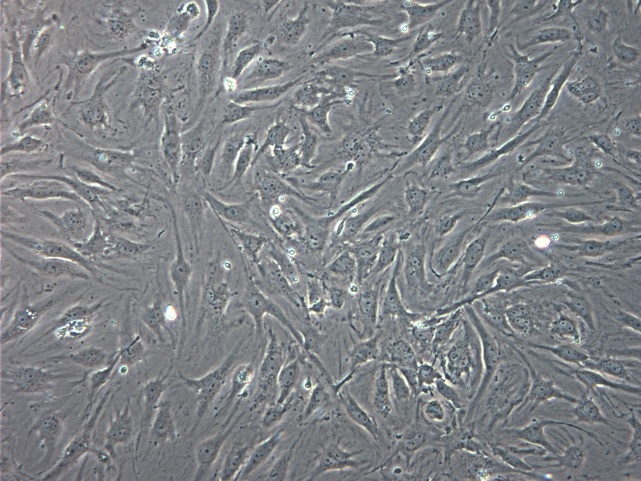

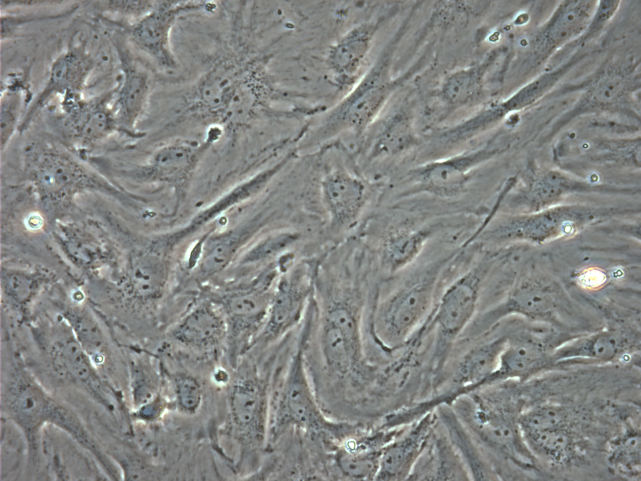

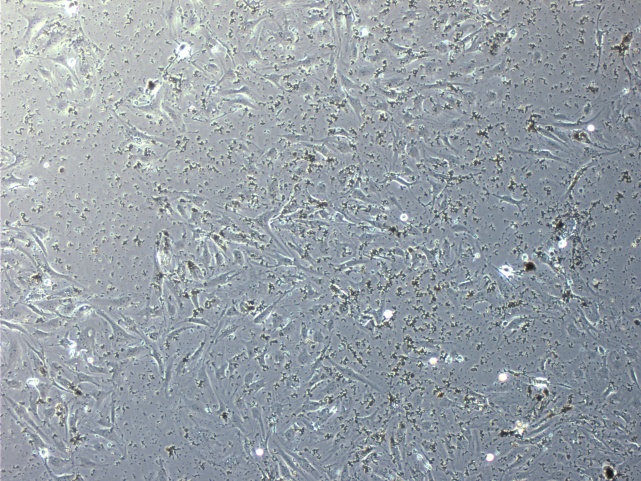

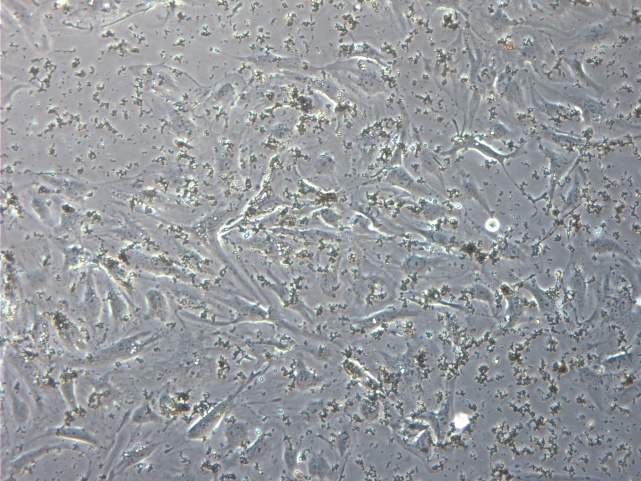

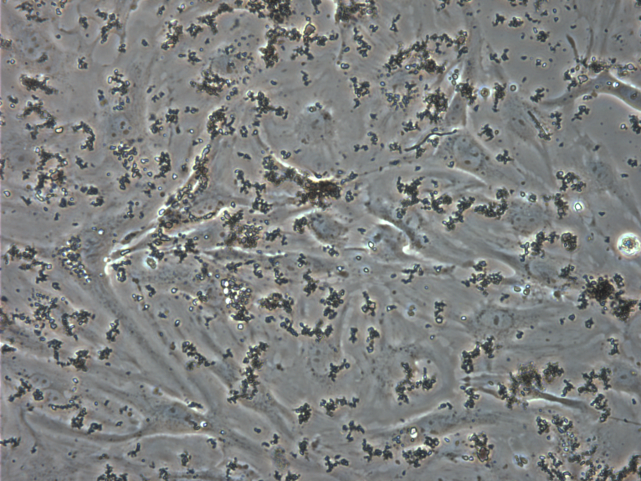

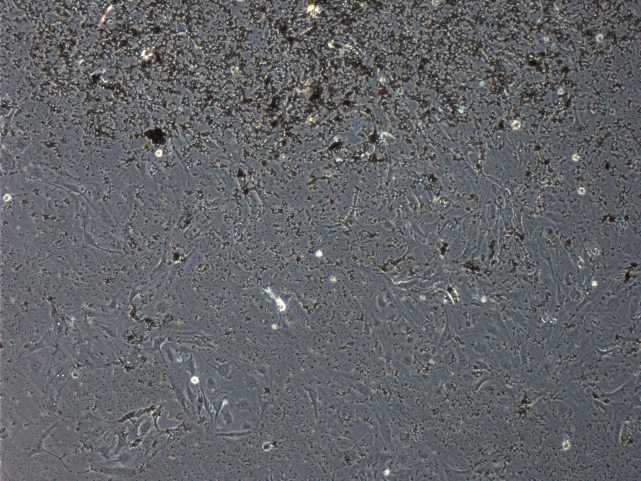

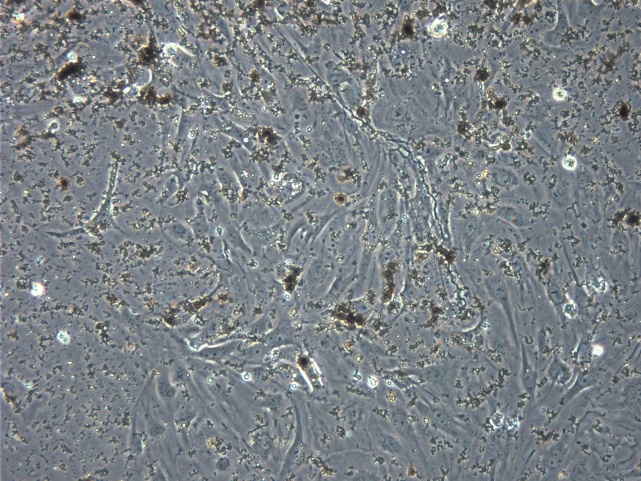

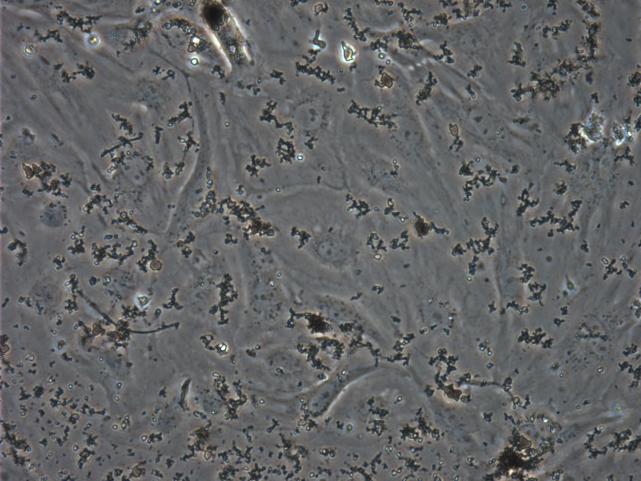


**Control**

**20 µg/ml**

**50 µg/ml**

**A**

**B**

Figure S3: MWNTs-NH_3_^+^ interaction with PBEC and the modified LDH assay. The uptake of MWNTs-NH_3_^+^ (20 and 50 μg/ml) into the PBEC monolayer was monitored by light microscopy. (A, B) Phase contrast photomicrographs showing the uptake pattern after 24 and 72 hours respectively. Interaction of MWNTs-NH_3_^+^ with the endothelial cells was apparent after 24 hours at both concentrations. The extent of interaction and uptake appeared higher after 72 hours. Scale bars = 100 um.

**
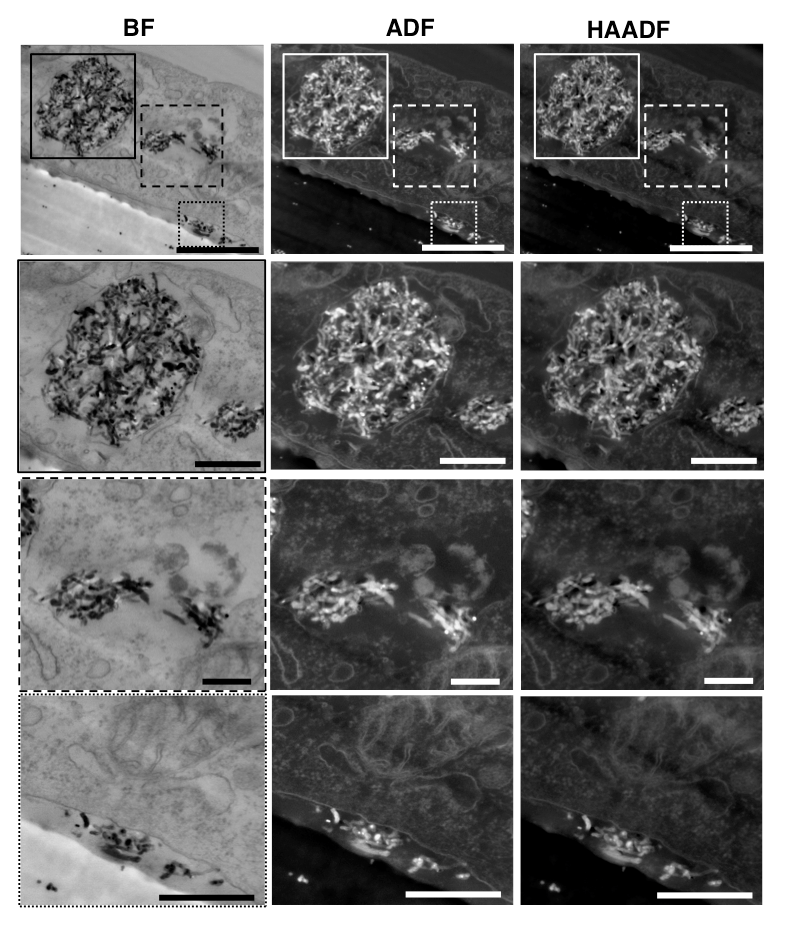
Figure S4: The uptake and transcytosis behavior of MWNTs across the PBEC monolayer.** Electron micrographs providing further evidence on the complete translocation of MWNTs-NH_3_^+^ across the *in vitro* BBB model. The MWNTs-NH_3_^+^ appeared on the basal side of endothelial cells after 24 hours of incubation. MWNT-NH_3_^+^ (20 µg/ml) were added to the apical chamber and incubated with the cells for 24 hours. Low voltage STEM imaging shows the MWNT-NH_3_^+^ within large endocytic vesicles **(solid square)** after 24 hours of incubation. MWNTs-NH_3_^+^ were also observed close to smaller vesicles in the abluminal side of PBEC **(dashed square)**. Some of the MWNT-NH_3_^+^ appeared outside the cells on the basal side **(dotted square)** with an intact plasma membrane indicating that the complete transcytosis of MWNT-NH_3_^+^ did not perturb the integrity of the plasma membrane. Scale bars from top to bottom; 1 μm, 500 nm, 500 nm and 500 nm.


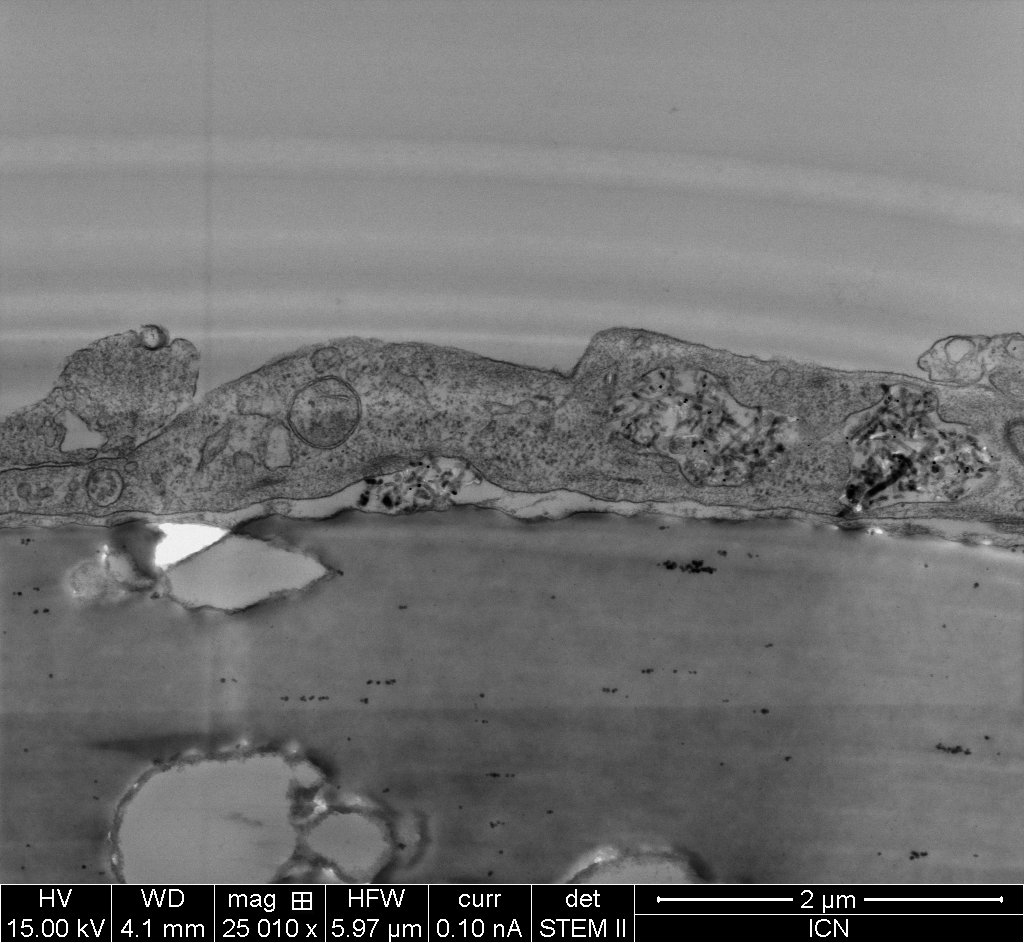

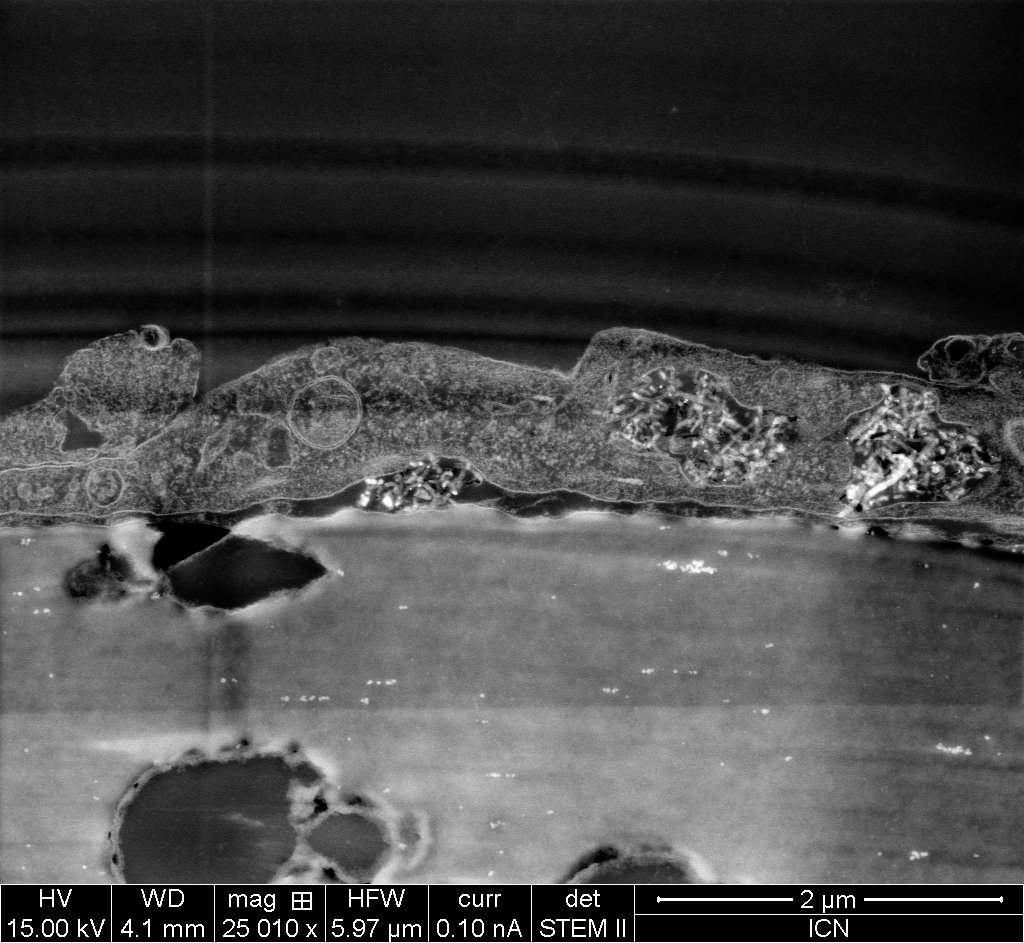

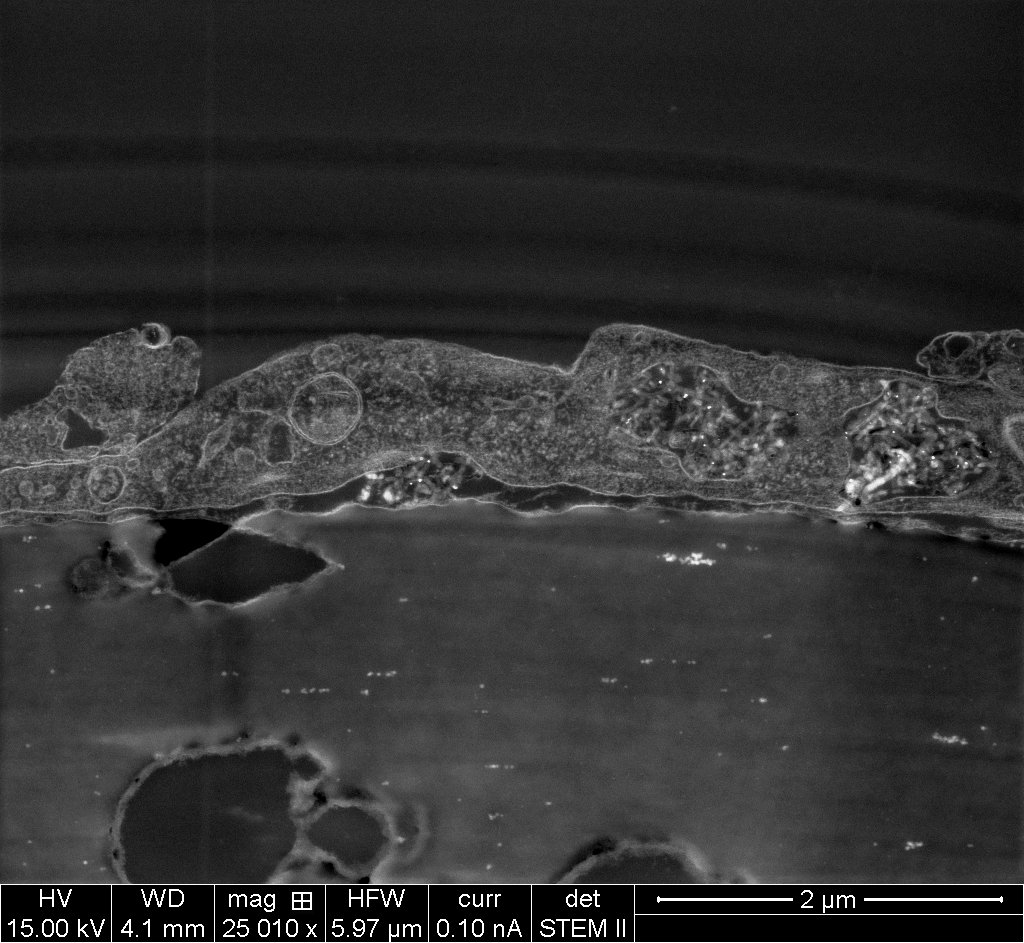

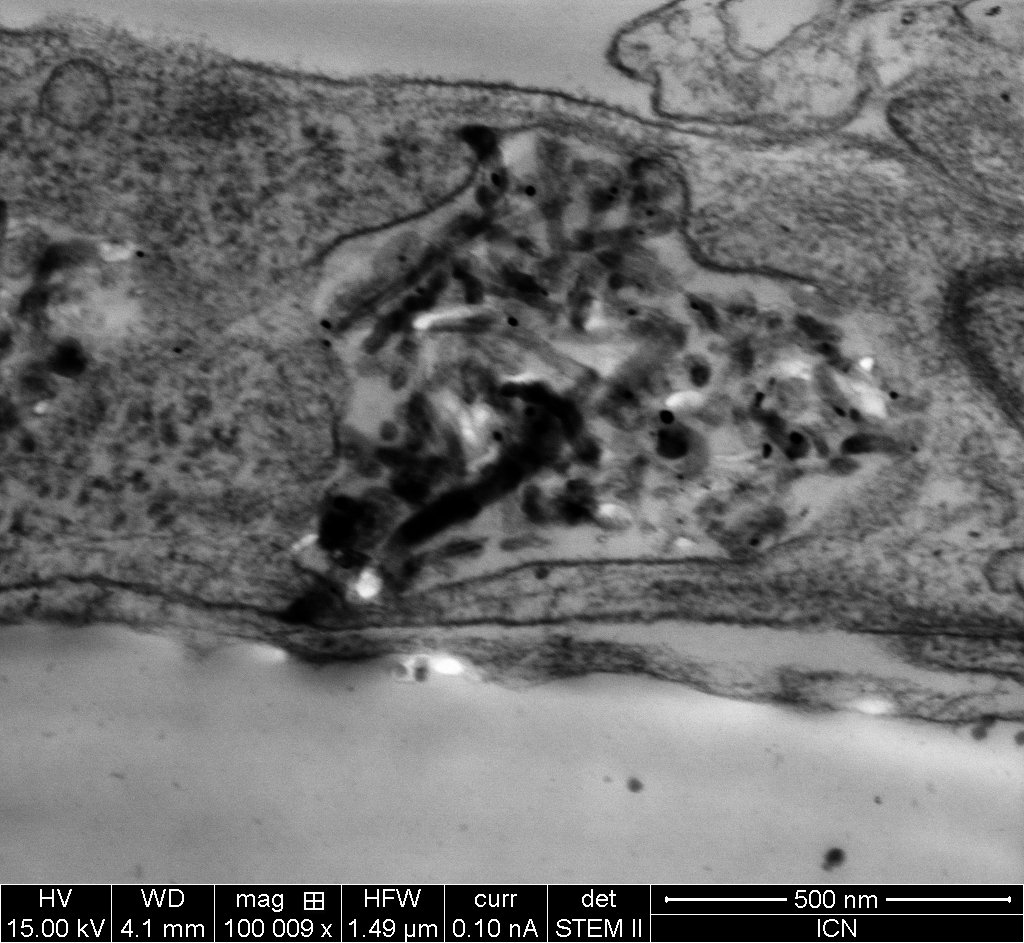

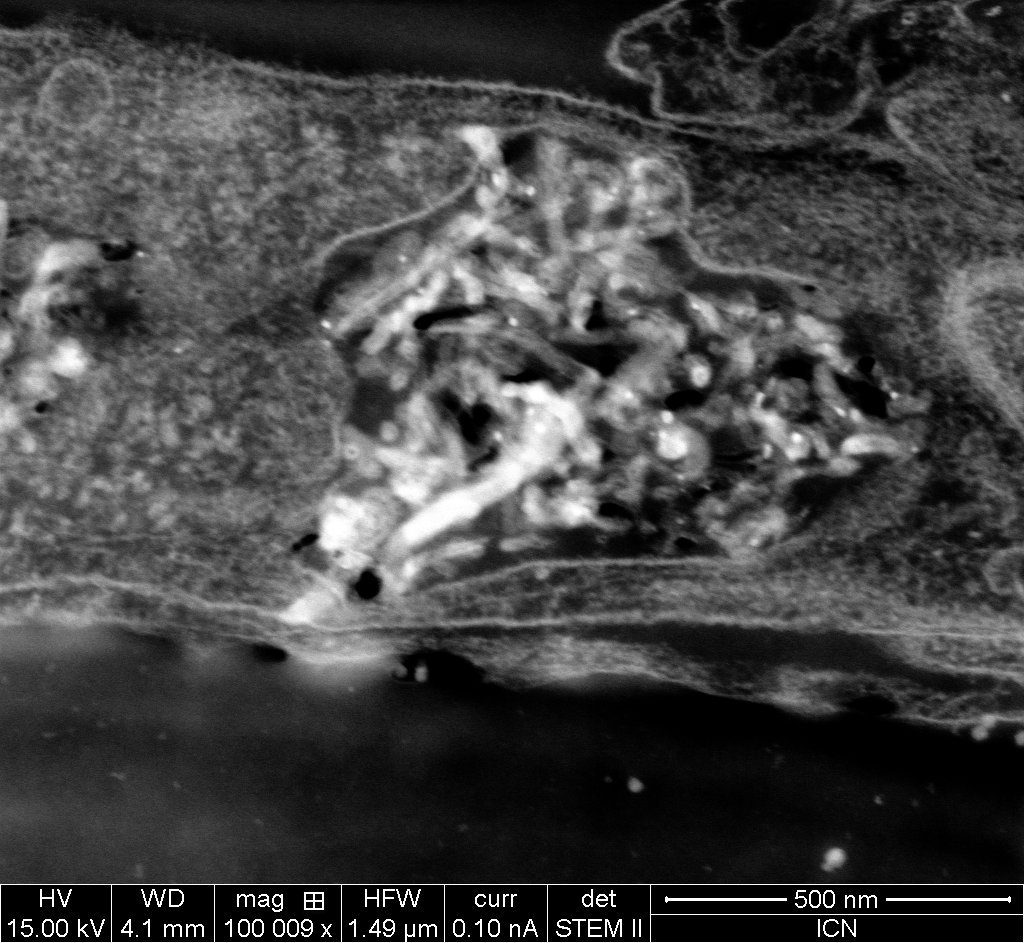

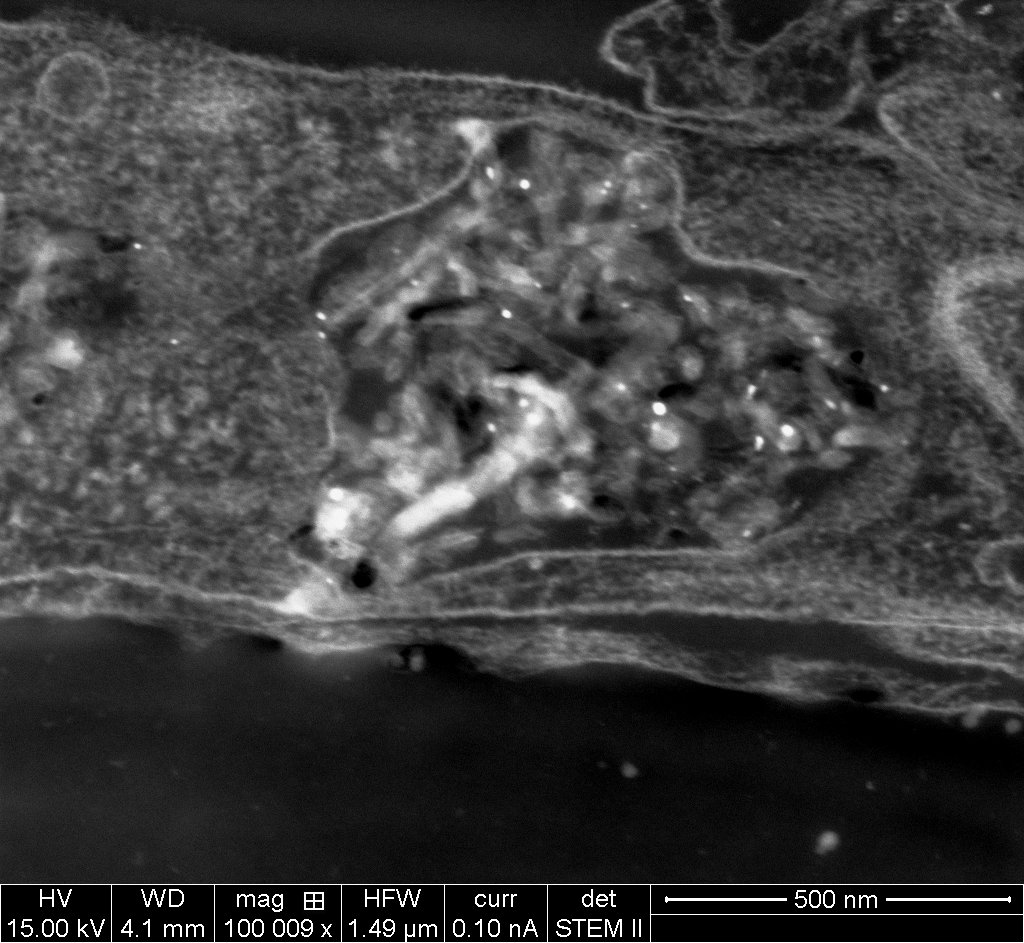

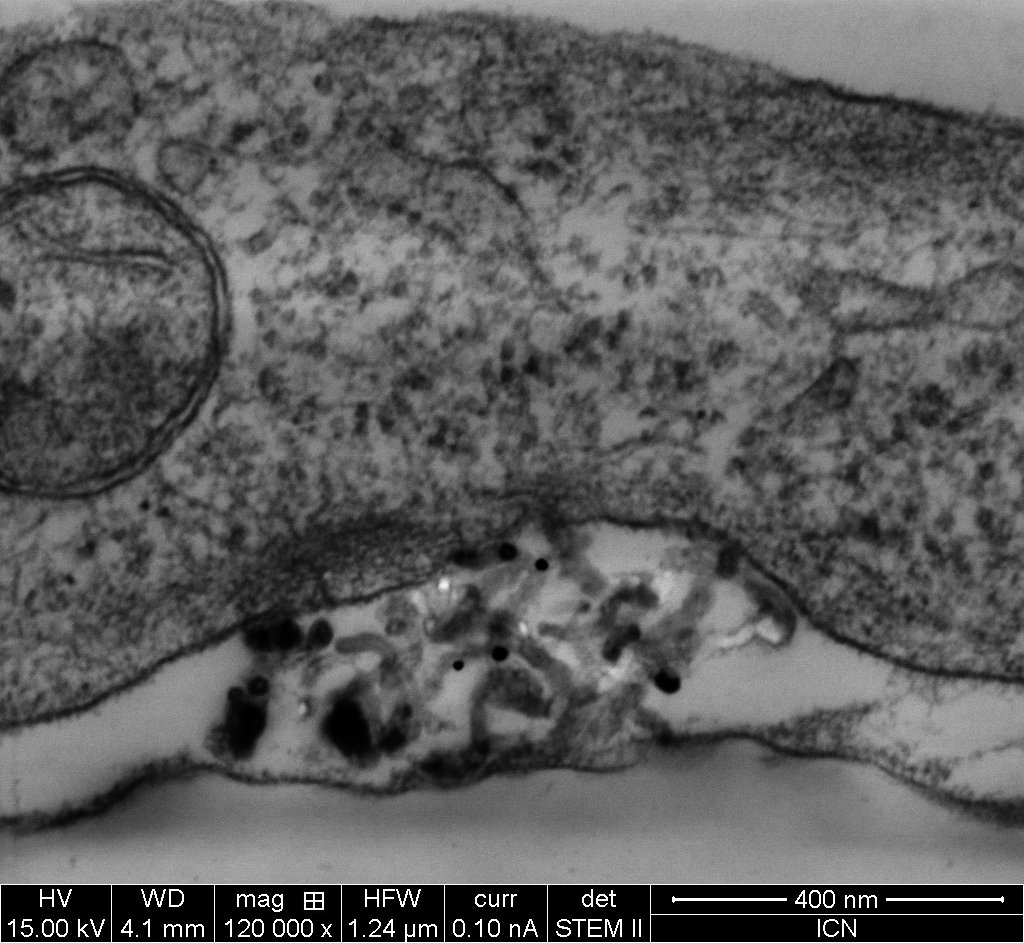

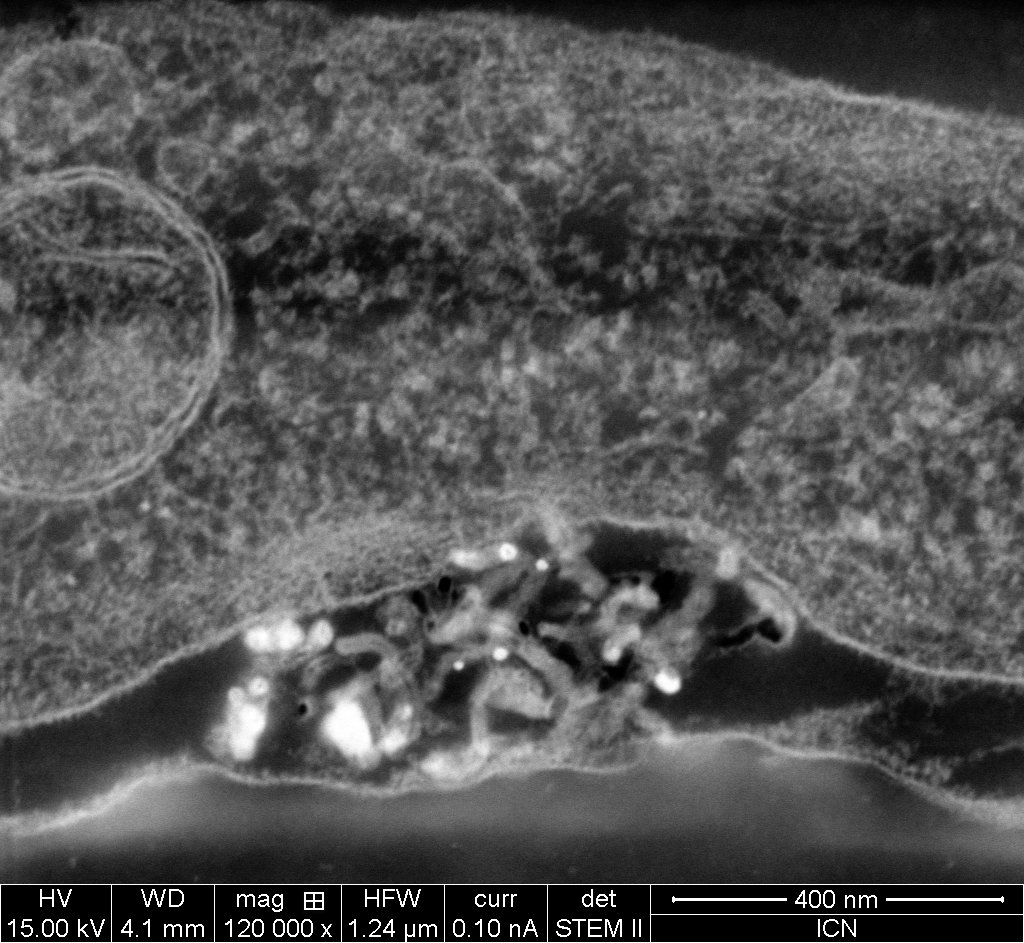

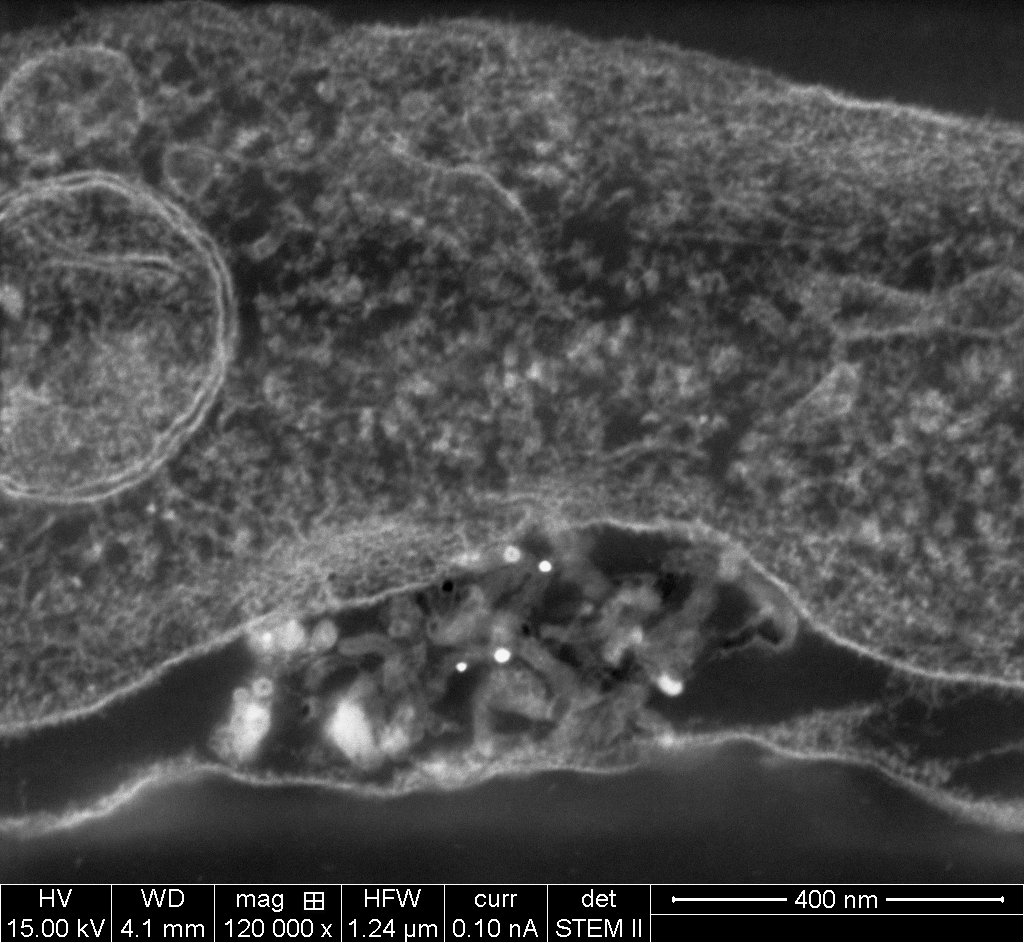


**A**

**(i)**

**(ii)**

**(iii)**


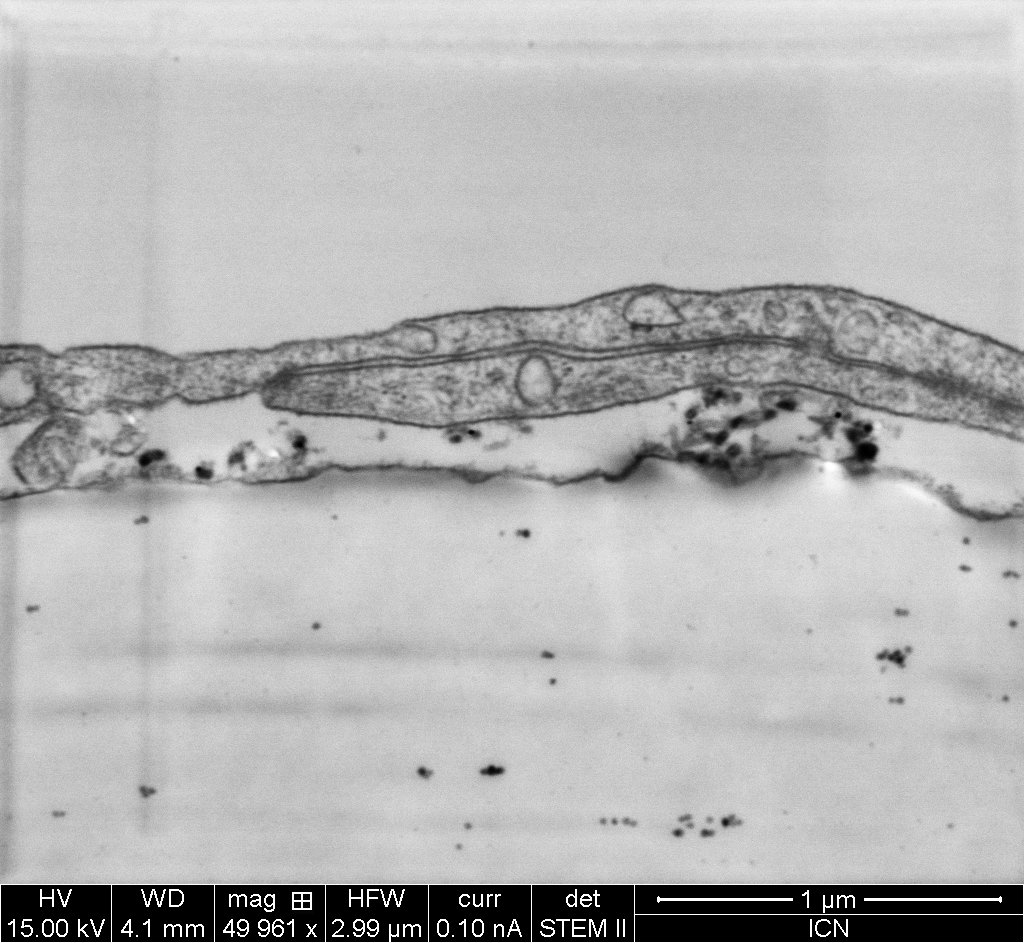

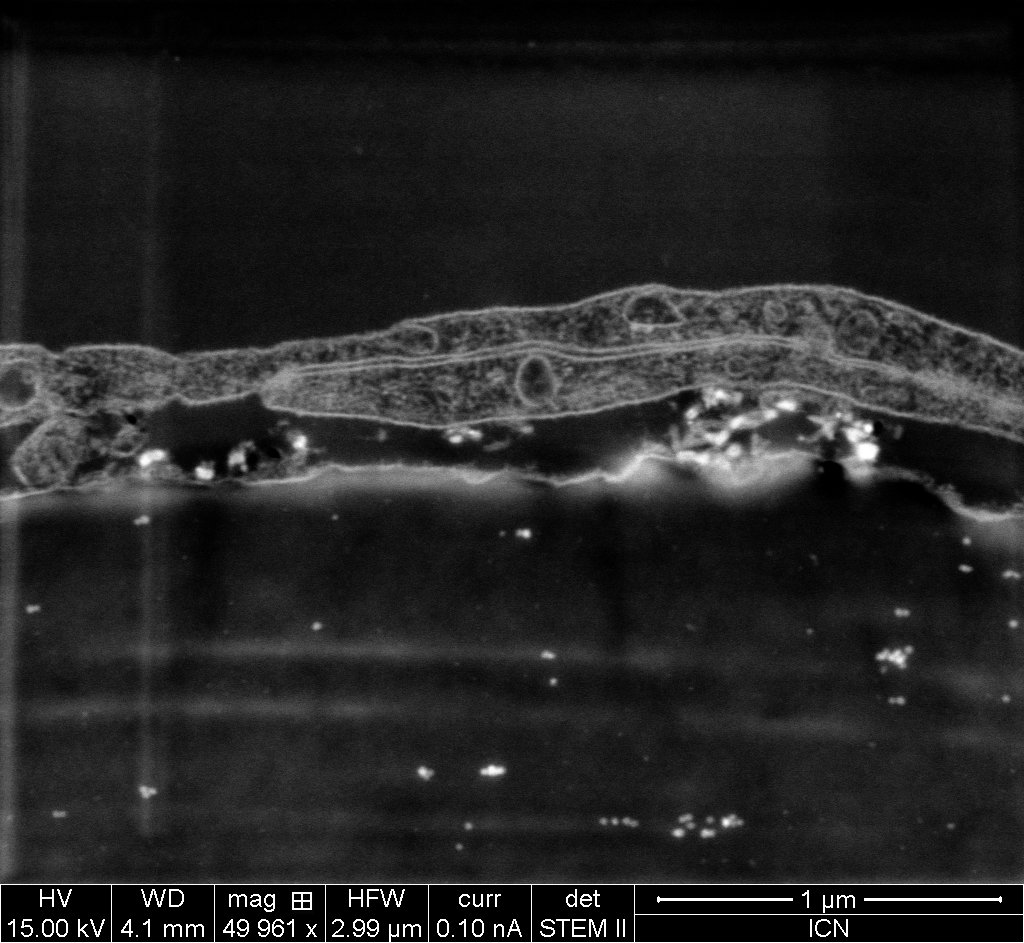

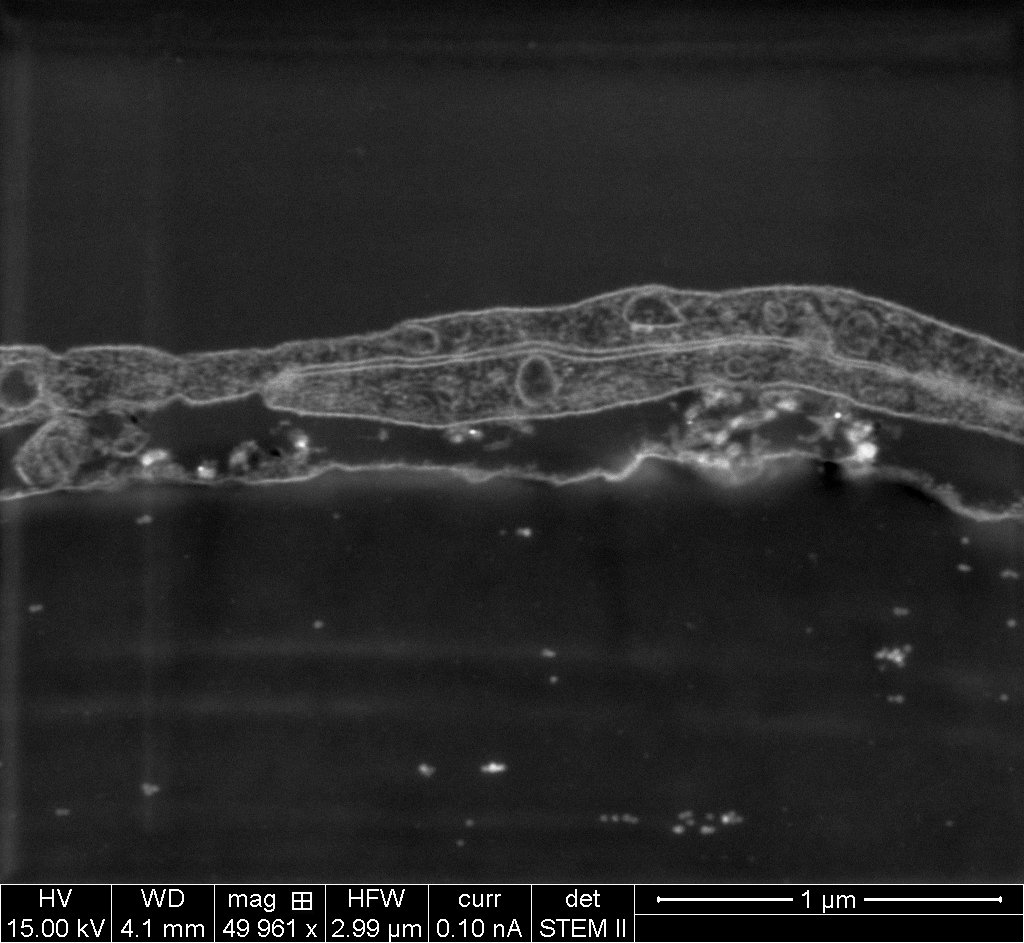

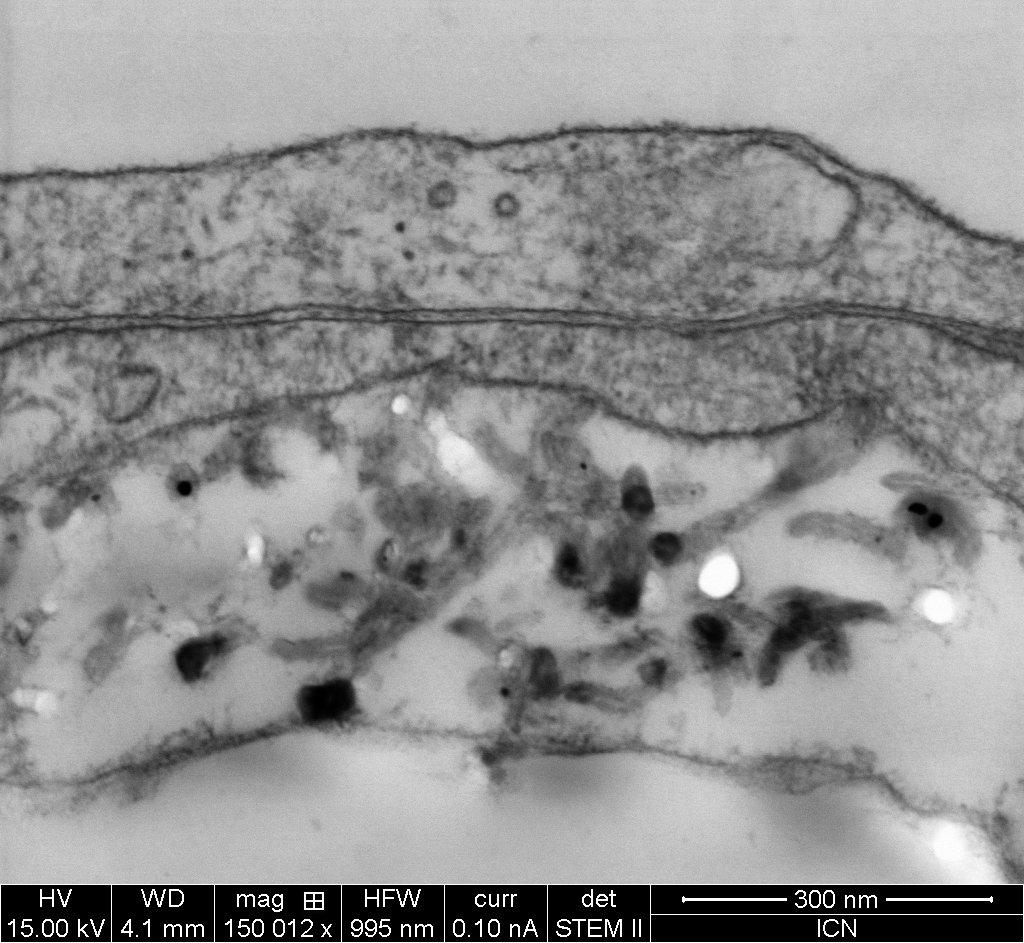

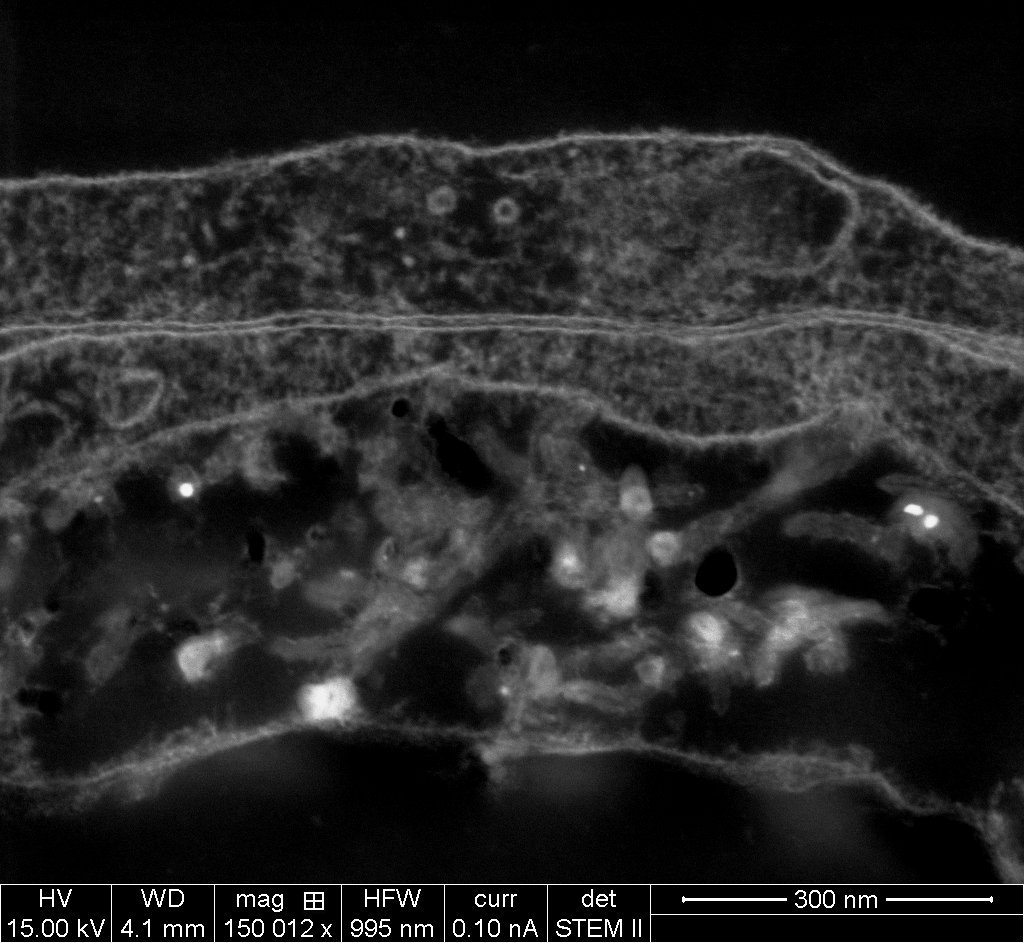

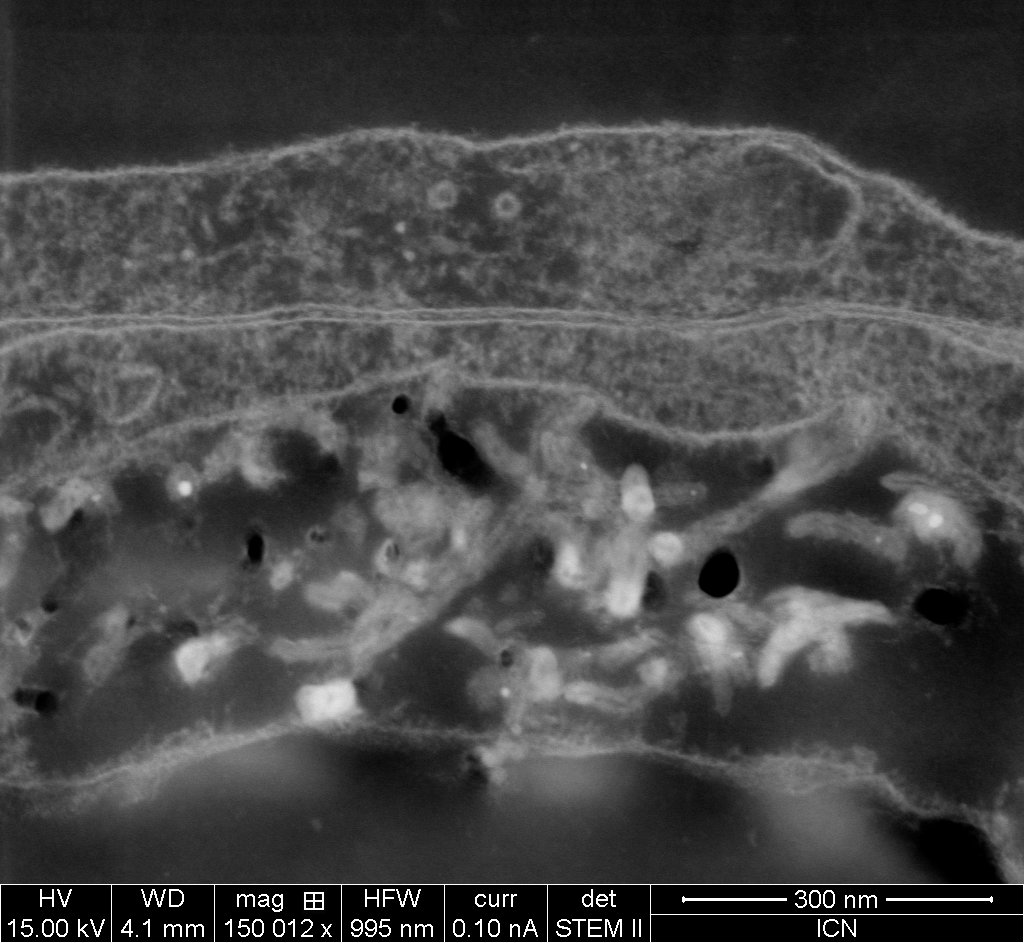


**B**

**(i)**

**(ii)**

**BF**

**ADF**

**HAADF**

Figure S5: Mechanism of uptake of MWNTs-NH_3_^+^ clusters across the PBEC monolayer. The low voltage STEM images show the apparent translocation of MWNTs-NH_3_^+^ from the apical to the basal side of the PBEC monolayer after 24 hours of incubation. (A i-iii) Electron micrographs showing evidence of membrane fusion of the MWNTs-NH_3_^+^-containing vesicles with the abluminal plasma membrane (Solid square). Also, the images show a cluster of MWNTs-NH_3_^+^ outside PBEC in the basal chamber, which appeared to have translocated across the PBEC monolayer after 24 hours of incubation (dashed square) (B i-ii) Electron micrographs providing further evidence on the complete translocation of MWNTs-NH_3_^+^ across the *in vitro* BBB model. The MWNTs-NH_3_^+^ appeared on the basal side of endothelial cells after 24 hours of incubation. Scale bars (A) from top to bottom, 2 μm, 500 nm and 400 nm (B) from top to bottom, 1 μm and 300 nm. Arrow heads indicate the tight junctions.

Figure S6: The interaction of DTPA-MWNTs and MWNTs-COOH with PBEC by TEM.


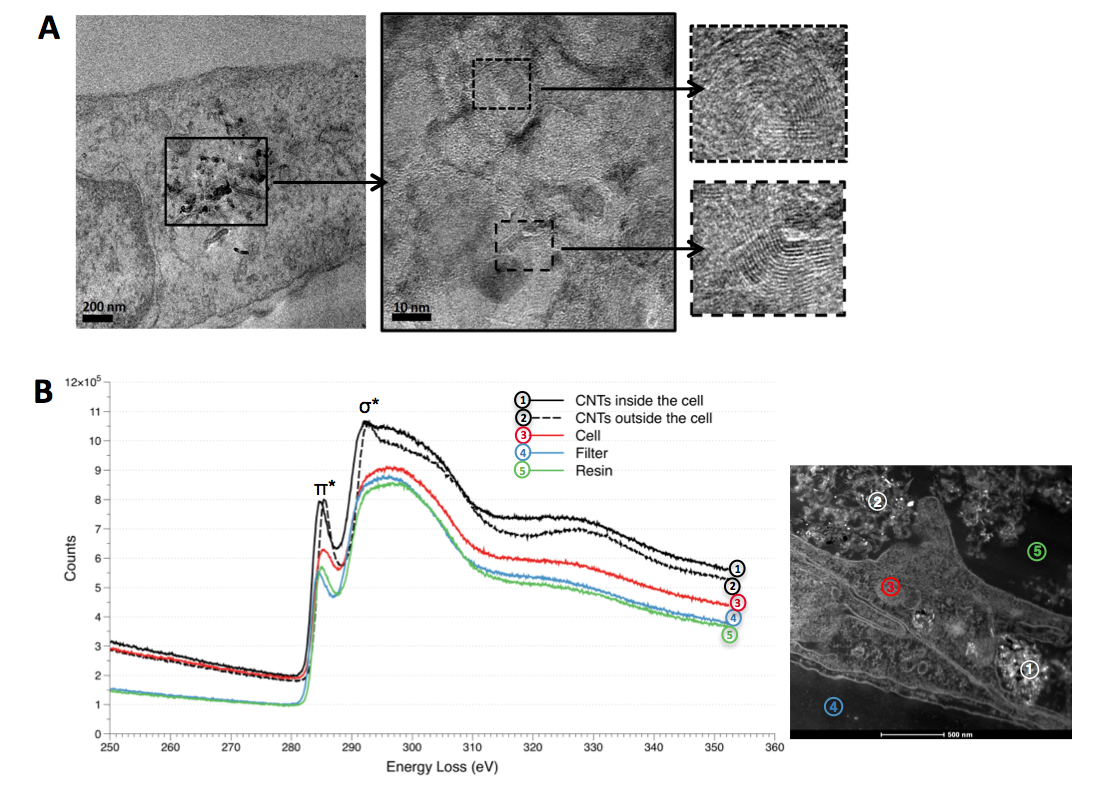


Figure S7: The integrity of the graphitic structure of MWNTs-NH_3_^+^ following uptake into endothelial cells. (A) High resolution TEM imaging showing the multi-walled structure of the MWNTs-NH_3_^+^. This confirms the integrity of the MWNTs-NH_3_^+^ within the cells, and also reflects the stability of these nanostructures against hydrolysing enzymes. The interlayer spacing between the walls is 0.34 nm (insets in a, far right). (B) Electron energy loss spectroscopy of MWNTs-NH_3_^+^ within and outside the endothelial cells. The HAADF-STEM image showed the measured points that included (1) MWNTs-NH_3_^+^ within the cells, (2) MWNTs-NH_3_^+^ outside the cells, (3) cell body, (4) polyester filter (used to grow cell on) and (5) the resin. The analysis of the carbon transitions of these points confirmed the graphitic structure of the MWNTs-NH_3_^+^ as compared to amorphous carbon detected in the cell body, resin or filters. The relative intensity of the π* peak from the MWNTs-NH_3_^+^ (points 1 and 2) was higher than the signals of the cell, filter or resin (points 3, 4 and 5). More importantly, the MWNTs spectra (points 1 and 2) showed a sharp σ* peak, characteristic of graphitic material, which confirms the stability of the MWNTs within the endothelial cells.


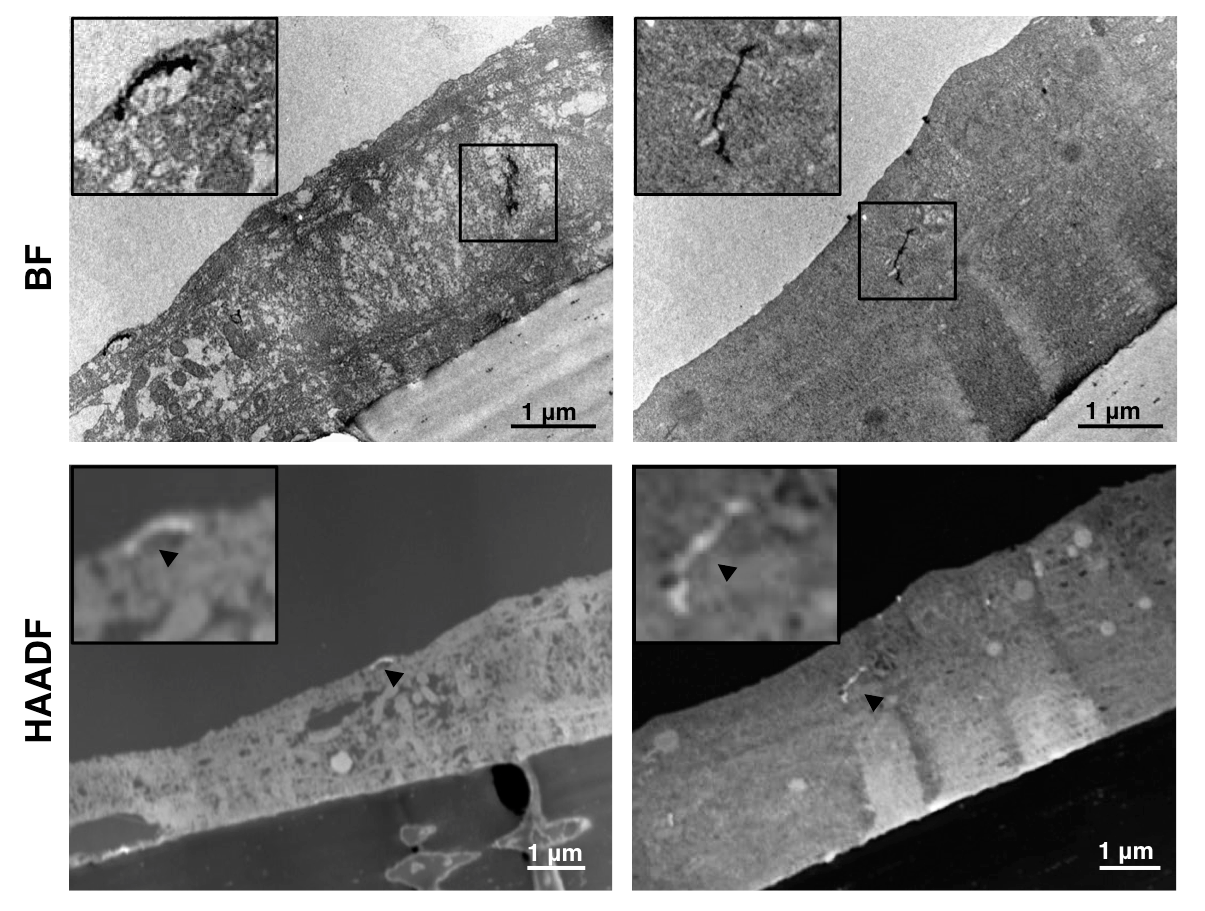


Figure S8: Uptake of MWNTs-NH_3_^+^ into astrocytes by HAADF. BF and HAADF electron micrographs showing the uptake of MWNTs-NH_3_^+^ into primary astoryctes after 24 hours of incubation with the PBEC monolayer. The MWNTs-NH_3_^+^ appeared as individual structures within the cytoplasm of the astrocytes with no evidence of vesicular uptake. The high contrast of the MWNTs-NH_3_^+^ in the HAADF images indicated the high electron density of the MWNTs-NH_3_^+^, which confirms the nature of the MWNTs-NH_3_^+^.

**
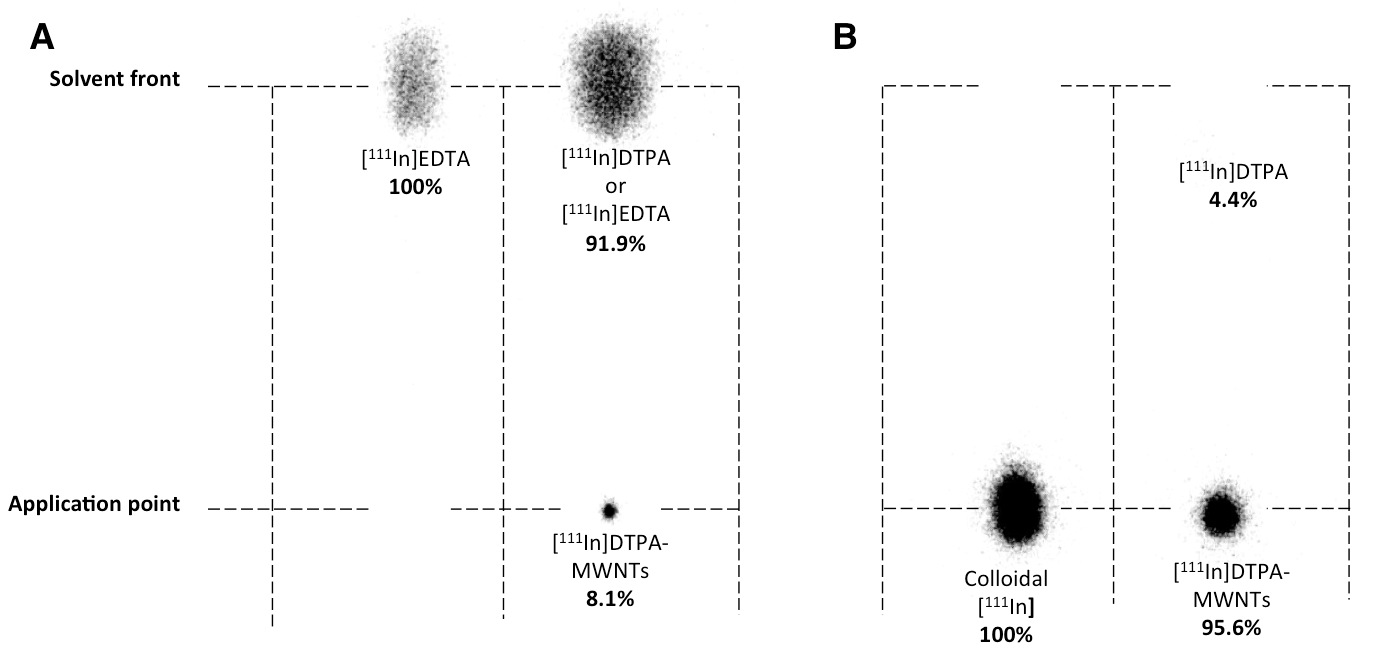
Figure S9: Radiolabeling studies of DTPA-MWNTs with ^111^Indium.** Chromatograms showing **(A)** radiolabelling efficiency and **(B)** degree of DTPA contamination. TLCs were generated using 0.1 M ammonium acetate buffer containing 50 mM EDTA **(A)** or 3.5% NH_3_:methanol (1:1) **(B)**. [^111^In]DTPA-MWNTs appear as an immobile spot while [^111^In]EDTA and/or [^111^In]DTPA appears as a mobile spot, representing the un-labelled fraction **(A)**. In **(B)**, only [^111^In]DTPA migrated to the solvent front because free ^111^In precipitates as colloidal ^111^In at alkaline pH. Only 4.4 % of the radio-labelled material could be attributed to [^111^In]DTPA rather than [^111^In]DTPA-MWNTs indicating the absence or very low content of free DTPA impurities.

**
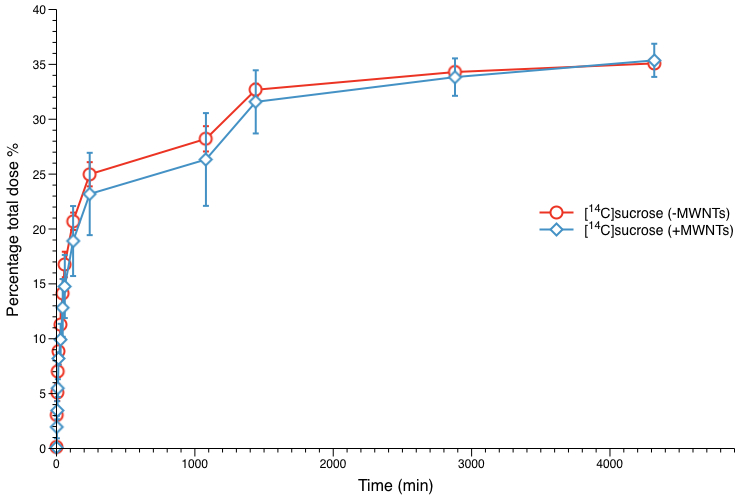
**

**Figure S10: The percentage of [^14^C]Sucrose permeating across the PBEC monolayer over 72 hours.** [^14^C]Sucrose (2.4x10^5^ dpm) was added at the apical chamber of 3.0 µm pore polycarbonate filters, with or without [^111^In]DTPA-MWNTs and incubated with PBECs at 37 °C. The radioactivity in the basal chamber was measured in 0.5 ml aliquots at different time points up to 72 hours. [^14^C]Sucrose was used in this experiment as a permeability marker to assess any damage to the tight junctions upon the addition of [^111^In]DTPA-MWNTs. The data show the permeation of [^14^C]sucrose across the PBEC monolayer over time, when incubated at 37 °C with or without [^111^In]DTPA-MWNTs reaching a maximum of 35.1 ± 0.5% and 35.4 ± 1.5%, respectively, with no statistical significance between the two conditions.

**
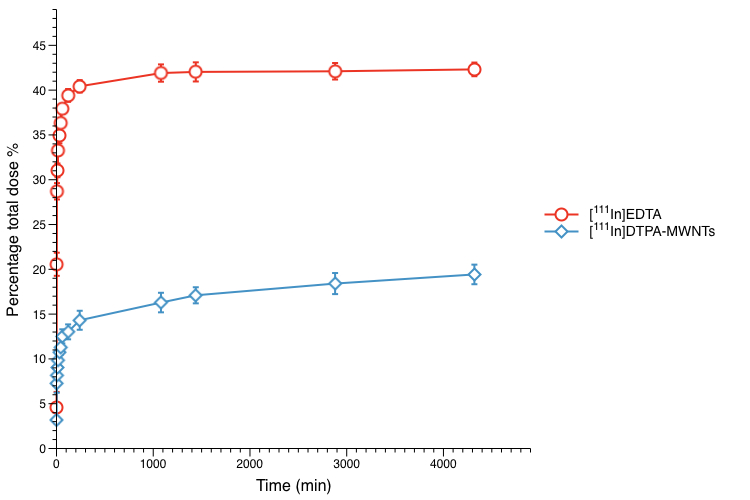
**

**Figure S11: The percentage of [^111^In]DTPA-MWNTs and [^111^In]EDTA permeating across the Transwell™ filter without cells.** [^111^In]DTPA-MWNTs (20 µg/ml) and [^111^In]EDTA were added to the apical chamber of 3.0 µm pore polycarbonate filters, and the radioactivity in the basal chamber was measured in 0.5 ml aliquots at different time points up to 72 hours. The results show an increase in the amount of [^111^In]DTPA-MWNTs and [^111^In]EDTA permeating over time reaching a maximum of 19 ± 1.1% and 42.3 ± 0.8% respectively after 72 hours.

**
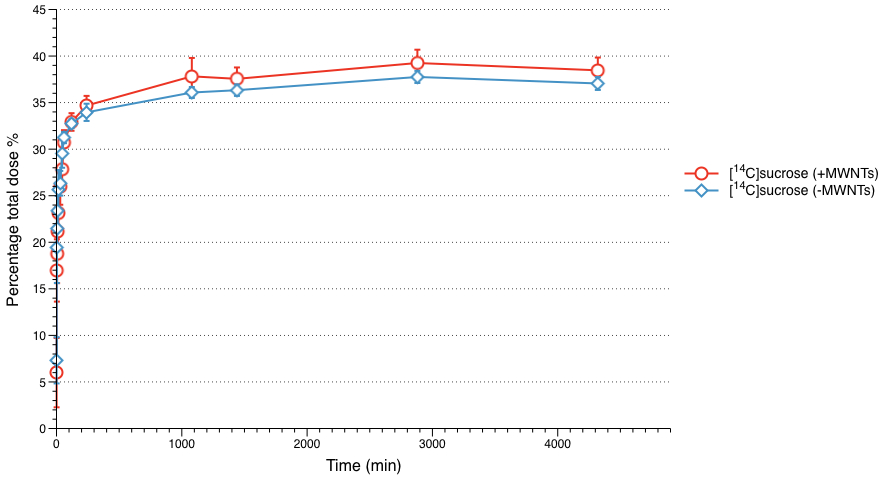
**

**Figure S12: The percentage of [^14^C]Sucrose permeating across the Transwell™ filter without cells.** [^14^C]Sucrose (2.4 x 10^5^ dpm) was added to the apical chamber of 3.0 µm pore polycarbonate filters with or without [^111^In]DTPA-MWNTs, and the radioactivity in the basal chamber was measured in 0.5 ml aliquots at different time points up to 72 hours. The results show an increase in the amount of [^14^C]sucrose permeating over time reaching a maximum of 38.5 ± 1.4% and 37.1 ± 0.7% in the presence and absence of [^111^In]DTPA-MWNTs, respectively, after 72 hours. No significant difference in the [^14^C]sucrose permeation was observed upon the addition of [^111^In]DTPA-MWNTs.


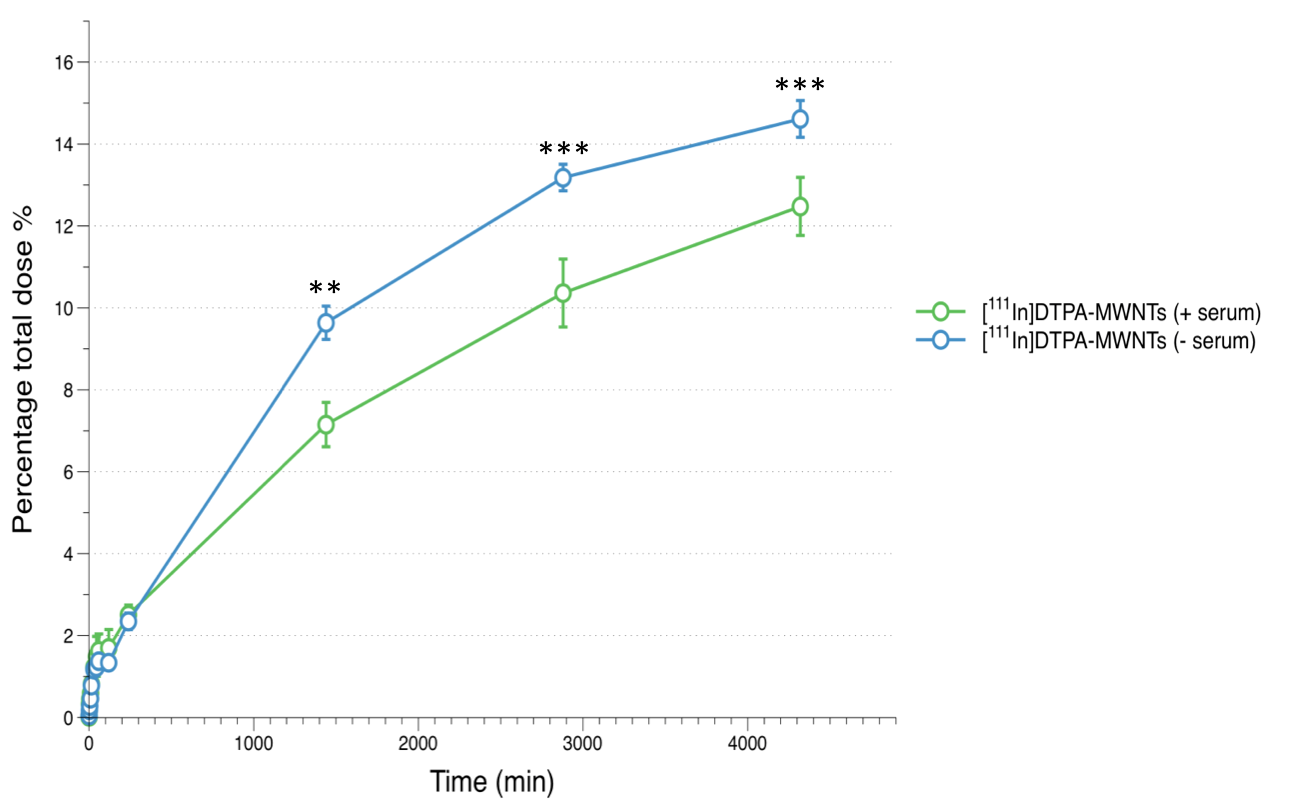


**Figure S13: The effect of serum on the transport of [^111^In]DTPA-MWNTs across PBEC.** [^111^In]DTPA-MWNTs (20 µg/ml) was added to the apical chamber of 3.0 µm pore polycarbonate filters, and the radioactivity in the basal chamber was measured in 0.5 ml aliquots at different time points up to 72 hours. The results show that the transport profile of [^111^In]DTPA-MWNTs was similar in the presence or absence of serum where the measured radioactivity increased in the basal chamber across the time points. The extent of transport was significantly higher in absence of serum after 24 (P<0.01), 48 hours and 72 hours (P<0.001) reaching a maximum of 14.6 ± 0.5 % and 12.5 ± 0.7 % in absence and presence of serum, respectively, after 72 hours (** P<0.01, *** P<0.001, n=3)

**Figure S14: The effect of MWNTs-NH3+ on TEER and the integrity of the PBEC monolayer**.

**Figure S15: Organ biodistribution and brain profile of [^111^In]DTPA-MWNTs in C57/Bl6 mice at various time points after systemic injection.** C57/Bl6 mice were injected with 50 μg of [^111^In]DTPA-MWNTs *via* the tail vein. Major organs were sampled at each time point after whole body perfusion with heparinised saline, followed by quantitative measurements of radioactivity by γ-scintigraphy. (A) the biodistribution of [^111^In]DTPA-MWNTs in the major organs after each time point. (B) Brain accumulation over time. Data is presented as % injected dose per gram tissue (% ID/g). Values are expressed as mean ± S.D (*n=3-4*).

**References**

[1] Pastorin G, Wu W, Wieckowski S, Briand JP, Kostarelos K, Prato M, et al. Double functionalisation of carbon nanotubes for multimodal drug delivery. Chem Commun. 2006:1182-4.

[2] Al-Jamal KT, Nunes A, Methven L, Ali-Boucetta H, Li S, Toma FM, et al. Degree of chemical functionalization of carbon nanotubes determines tissue distribution and excretion profile. Angewandte Chemie. 2012;51:6389-93.

[3] Nunes A, Bussy C, Gherardini L, Meneghetti M, Herrero MA, Bianco A, et al. In vivo degradation of functionalized carbon nanotubes after stereotactic administration in the brain cortex. Nanomedicine (Lond). 2012;7:1485-94.

[4] Smith BW, Luzzi DE. Electron irradiation effects in single wall carbon nanotubes. J Appl Phys. 2001;90:3509-15.

[5] Cellot G, Cilia E, Cipollone S, Rancic V, Sucapane A, Giordani S, et al. Carbon nanotubes might improve neuronal performance by favouring electrical shortcuts. Nat Nanotechnol. 2009;4:126-33.
